# Sequence analysis of percent G+C fraction libraries of human faecal bacterial DNA reveals a high number of *Actinobacteria*

**DOI:** 10.1186/1471-2180-9-68

**Published:** 2009-04-08

**Authors:** Lotta Krogius-Kurikka, Anna Kassinen, Lars Paulin, Jukka Corander, Harri Mäkivuokko, Jarno Tuimala, Airi Palva

**Affiliations:** 1 Department of Basic Veterinary Sciences, Faculty of Veterinary Medicine, PO Box 66, FI-00014 University of Helsinki, Finland; 2 DNA Sequencing Laboratory, Institute of Biotechnology, University of Helsinki, Finland; 3 Department of Mathematics, Åbo Akademi University, Finland; 4 Danisco Innovation, Kantvik, Finland; 5 CSC – Scientific Computing Ltd, Espoo, Finland; 6 The Finnish Red Cross, Blood Service, Helsinki, Finland

## Abstract

**Background:**

The human gastrointestinal (GI) tract microbiota is characterised by an abundance of uncultured bacteria most often assigned in phyla *Firmicutes *and *Bacteroidetes*. Diversity of this microbiota, even though approached with culture independent techniques in several studies, still requires more elucidation. The main purpose of this work was to study whether the genomic percent guanine and cytosine (%G+C) -based profiling and fractioning prior to 16S rRNA gene sequence analysis reveal higher microbiota diversity, especially with high G+C bacteria suggested to be underrepresented in previous studies.

**Results:**

A phylogenetic analysis of the composition of the human GI microbiota of 23 healthy adult subjects was performed from a pooled faecal bacterial DNA sample by combining genomic %G+C -based profiling and fractioning with 16S rRNA gene cloning and sequencing. A total of 3199 partial 16S rRNA genes were sequenced. For comparison, 459 clones were sequenced from a comparable unfractioned sample. The most important finding was that the proportional amount of sequences affiliating with the phylum *Actinobacteria *was 26.6% in the %G+C fractioned sample but only 3.5% in the unfractioned sample. The orders *Coriobacteriales, Bifidobacteriales *and *Actinomycetales *constituted the 65 actinobacterial phylotypes in the fractioned sample, accounting for 50%, 47% and 3% of sequences within the phylum, respectively.

**Conclusion:**

This study shows that the %G+C profiling and fractioning prior to cloning and sequencing can reveal a significantly larger proportion of high G+C content bacteria within the clones recovered, compared with the unfractioned sample in the human GI tract. Especially the order *Coriobacteriales *within the phylum *Actinobacteria *was found to be more abundant than previously estimated with conventional sequencing studies.

## Background

The gastrointestinal (GI) microbiota is considered to play an important role in human health and disease via essential metabolic, trophic and protective functions in the host [1]. Since the majority of the GI bacteria are uncultivable, molecular biology methods are needed to reveal the detailed composition, diversity and specific role of this complex microbial community [2]. The bacterial groups most often detected in molecular studies of the healthy human GI tract are phyla *Firmicutes *(especially *Clostridium *clusters XIVa and IV), *Bacteroidetes*, *Proteobacteria, Actinobacteria, Fusobacteria *and *Verrucomicrobia *[3]. The predominant microbiota in adults is considered rather stable and host-specific [4,5], but gender, geographic origin, age [6,7], and host genotype [8] may influence its composition. Furthermore, alterations within an individual's environmental factors, such as diet [9] and dietary supplements [10], intestinal health status [11] and antibiotics [12], may also have a substantial effect on the intestinal microbiota. Therefore, as a reference to altered conditions, knowledge of the characteristics of a healthy intestinal microbiota is essential.

The proportional amounts of bacterial phyla detected in studies on the GI tract microbiota depend on both the sample handling and DNA extraction methods applied [13] and the analysis [14]. Recent metagenomic and pyrosequencing studies on the human intestinal microbiota highlight the potential amount of the yet undiscovered diversity of phylotypes and reshape the porportional abundances of the detected phyla, revealing e.g. a higher abundance of *Actinobacteria *than previously estimated [14-16]. However, the conventional 16S rRNA gene cloning and sequencing is still a valuable method, since it gives a relatively high taxonomic resolution due to longer read length [12] and can be targeted to a phylogenetically relevant gene (16S rRNA gene) in comparison with the metagenomic approach. Furthermore, the clone library obtained serves as a valuable reference for possible future use. To enhance the recovery of phylotypes in bacterial community samples, the genomic %G+C content -based profiling and fractioning of DNA can be used [17-20].

In a previous study comparing patients suffering from irritable bowel syndrome (IBS) with healthy volunteers, the faecal DNA of 23 healthy donors was pooled and %G+C profiled and three selected fractions, covering 34% of the fractioned DNA, were cloned and sequenced [21]. With the aim to comprehensively elucidate the bacterial phylotype diversity of the GI microbiota of healthy subjects, the remaining seven %G+C fractions were cloned and sequenced in this study, to represent the scale of bacterial genomic %G+C content ranging from 25% to 75% [22]. For methodological comparison, a clone library from unfractioned pooled faecal DNA samples of the same study subjects was constructed. The results provide more detailed insight into the human GI microbiota especially in the context of the diversity of high %G+C bacteria, i.e. *Actinobacteria*.

## Results

### Percent guanine plus cytosine -profiling, cloning and sequencing

**Figure 1:**
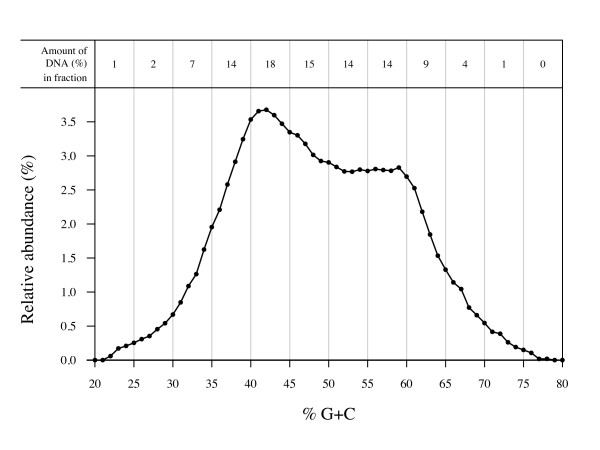
**Percent guanine plus cytosine profile of intestinal microbial genomic DNA pooled from 23 healthy subjects**. The amount of DNA is indicated as relative absorbance (%) and the area under the curve is used for calculating the proportional amount of DNA in the separate fractions (modified from Kassinen et al. [21]).

To analyse the diversity of the healthy human intestinal microbiota, a %G+C profiled and fractionated (Figure 1) pooled faecal bacterial DNA sample of 23 individuals was cloned, and the partial 16S rRNA genes were sequenced. The previously published 976 sequences from three %G+C fractions (%G+C 25–30, 40–45 and 55–60) [21] were combined with the 2223 new sequences cloned in this study (%G+C fractions 30–35, 35–40, 45–50, 50–55, 60–65, 65–70 and 70–75) for phylogenetic and statistical analyses of the complete %G+C profile ranging from 25% G+C to 75% G+C (Figure 1, Table 1). Altogether, 3199 sequences encompassing approximately 450 bp from the 5'-end of the 16S rRNA gene, covering two variable areas V1 and V2, were sequenced from all clones from the fractioned sample. For comparison, 459 clones were sequenced from an unfractioned pooled faecal bacterial DNA sample originating from the same individuals.

**Table 1:** Characteristics of the sequence libraries.

Library(s)	Sequences (no.)	OTUs (no.)^a^	%G+C^b^	Singletons (no.)	Coverage^c^
Fr G+C 25–30%	319	91	51.5	43	87
Fr G+C 30–35%	350	94	52.6	48	86
Fr G+C 35–40%	313	93	53.4	50	84
Fr G+C 40–45%	346	119	53.9	67	81
Fr G+C 45–50%	316	112	56.0	62	80
Fr G+C 50–55%	292	62	58.1	22	93
Fr G+C 55–60%	311	45	62.1	22	93
Fr G+C 60–65%	303	64	61.7	26	91
Fr G+C 65–70%	362	130	57.6	65	82
Fr G+C 70–75%	287	116	55.5	67	77
Fr G+C 25–75%^d^	3199	455	56.2	180	94
Unfractioned	459	131	53.6	66	86

a. The number of OTUs determined with DOTUR using 98% similarity criterion [53]

b. Average %G+C content of the partial 16S rRNA gene sequences

c. Coverage according to Good [23]

d. The combined G+C fractions

### Determination of operative taxonomic units and library coverage

**Figure 2:**
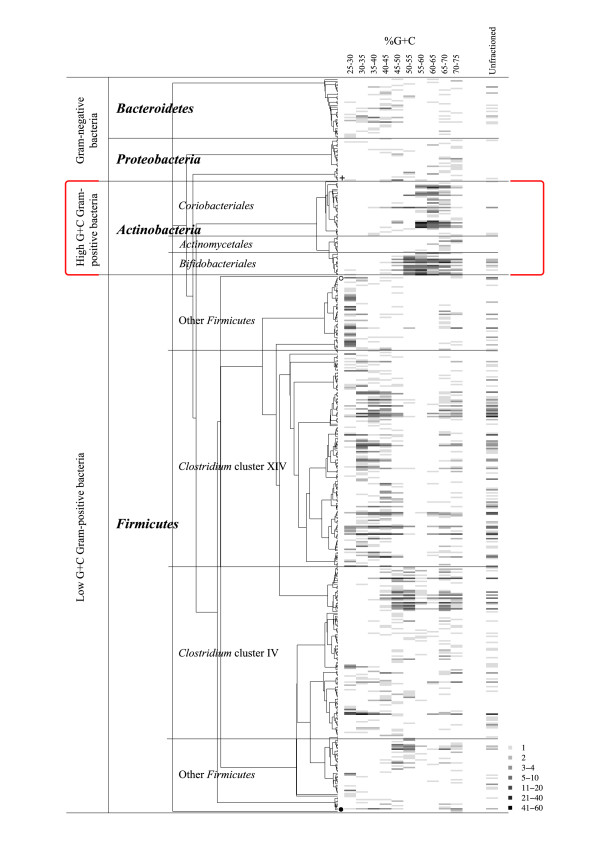
**Cladogram and abundance plot of the phylogenetic affiliation of the 481 OTUs comprising 3658 sequences**. The grey scale indicates the OTU abundance in the %G+C fraction libraries and in the unfractioned library. *Actinobacteria *are abundant in the high %G+C fractions (in square brackets). *Acidobacteria *and *Verrucomicrobia *phylotypes are denoted with a cross. A phylotype having 79% affiliation with *Proteobacteria *is indicated with an open circle. Phylotypes having 100% affiliation with *Cyanobacteria*, and 94% affiliation with TM7 with RDPII Classifier [55] are indicated with a black sphere.

The quality-checked 3199 sequences from the combined fractioned sample libraries represented 455 operative taxonomic units (OTUs), and the 459 sequences from the unfractioned sample represented 131 OTUs with a 98% similarity criterion (Table 1). All novel OTUs with less than 95% sequence similarity to public sequence database entries were further sequenced to near full-length (Additional file 1). The coverages of the individual clone libraries of the fractioned sample ranged from 77% to 93%, while the coverage for the unfractioned sample was 86% [23] (Table 1). Compared with other fractions, the fractions %G+C 50–55, 55–60 and 60–65 had low OTU numbers and few singletons, resulting in high Good's coverage values. The combined sequences from the fractioned and unfractioned samples clustered into 481 OTUs (Figure 2).

### Phylogenetic analysis and sequence affiliation

**Figure 3:**
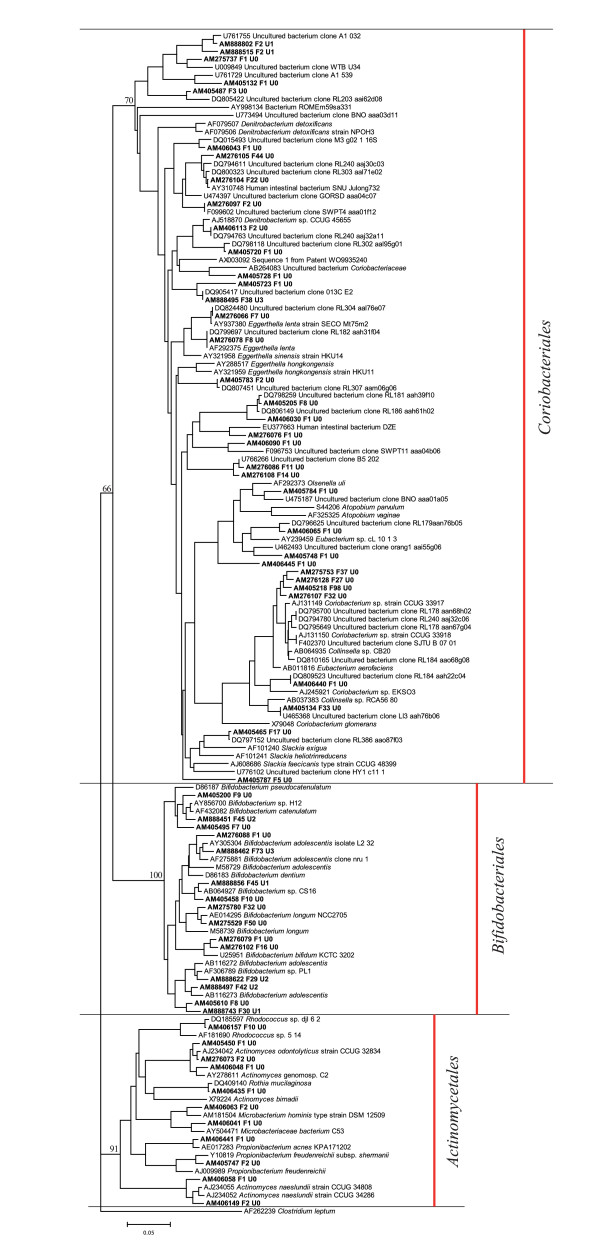
**Phylogenetic tree of actinobacterial OTUs in the fraction libraries and in the unfractioned library**. The amount of sequences in the representative OTUs are denoted after the letter F (fractioned sequence libraries) and U (unfractioned library). Bootstrap values are percentages of 100 resamplings and the scale bar represents 0.05 substitutions per nucleotide position.

When the sequence data from the fractioned clone libraries were combined, the majority of the sequences were assigned to the phyla *Firmicutes *(68.5%), *Actinobacteria *(26.6%), *Bacteroidetes *(3.1%) and *Proteobacteria *(1.3%) (Figure 2, Table 2, Additional file 1). *Clostridium *clusters IV and XIV were the most abundant *Firmicutes *represented by 23.5% and 33.0% of the sequences, respectively. The 65 actinobacterial phylotypes consisted of the orders *Bifidobacteriales*, *Coriobacteriales *and *Actinomycetales *accounting for 12.4%, 13.4% and 0.8% of the sequences, respectively (Figure 3, Table 2).

**Table 2:** Phylogenetic affiliation of OTUs and sequences of the %G+C fractioned libraries and the unfractioned library.

Library	Fractioned G+C 25–75%		Unfractioned	
Group	OTUs n (%)	Sequences n (%)	OTUs n (%)	Sequences n (%)

Phylum *Firmicutes*	323 (71.0)	2190 (68.5)	113 (86.3)	428 (93.2)
*Clostridium *cluster IV	107 (23.5)	753 (23.5)	36 (27.5)	131 (28.5)
*Clostridium *cluster XIV	131 (28.8)	1057 (33.0)	52 (39.7)	233 (51.0)
*Enterococcaceae*	2 (0.4)	5 (0.2)	0 (0)	0 (0)
*Lactobacillaceae*	4 (0.9)	34 (1.1)	0 (0)	0 (0)
*Staphylococcaceae*	2 (0.4)	2 (0.1)	0 (0)	0 (0)
*Streptococcaceae*	6 (1.3)	20 (0.6)	2 (1.5)	5 (1.1)
Other *Firmicutes*	71 (15.6)	311 (9.7)	22 (16.8)	58 (12.6)
Phylum *Actinobacteria*	65 (14.3)	851 (26.6)	8 (6.1)	16 (3.5)
*Actinomycetales*	10 (2.2)	24 (0.8)	0 (0)	0 (0)
*Bifidobacteriales*	17 (3.7)	398 (12.4)	5 (3.8)	11 (2.4)
*Coriobacteriales*	38 (8.4)	429 (13.4)	3 (2.3)	5 (1.1)
Phylum *Bacteroidetes*	37 (8.1)	99 (3.1)	8 (6.1)	13 (2.8)
Phylum *Proteobacteria*	24 (5.3)	42 (1.3)	1 (0.8)	1 (0.2)
*Alphaproteobacteria*	3 (0.7)	6 (0.2)	0 (0)	0 (0)
*Betaproteobacteria*	9 (2.0)	16 (0.5)	0 (0)	0 (0)
*Deltaproteobacteria*	5 (1.1)	11 (0.3)	0 (0)	0 (0)
*Gammaproteobacteria*	7 (1.5)	9 (0.3)	1 (0.8)	1 (0.2)
Other phyla^a^	6 (1.3)	17 (0.5)	1 (0.8)	1 (0.2)
Sum	455	3199	131	459

a. Affiliation with *Acidobacteria*, *Cyanobacteria*, TM7 and *Verrucomicrobia*

The distribution of phyla within the individual clone libraries of the fractioned sample revealed that *Firmicutes *settled mostly in the lower %G+C content portion of the profile, whereas *Actinobacteria *were found in the fractions with a %G+C content ranging from 50% to 70% (Figure 2, Additional file 1). Prominent phylotypes had a seemingly broader distribution across %G+C fractions. In the fractions having %G+C content above 65%, a bias was observed, i.e. a decrease in high G+C *Actinobacteria *and an increase in low G+C *Firmicutes*. The three OTUs with the highest number of sequences fell into the *Clostridium *clusters XIVa and IV, representing the species *Eubacterium rectale *(cluster XIVa), *Faecalibacterium prausnitzii *(cluster IV) and *Ruminococcus bromii *(cluster IV) with over 98.7% sequence similarity.

Within the phylum *Actinobacteria*, the most abundant *Coriobacteriales *phylotypes (6 OTUs) according to the number of representative clones (228 clones) affiliated with *Collinsella *sp. (*C. aerofaciens*). The remainder represented *Atopobium *sp., *Denitrobacterium *sp., *Eggerthella *sp., *Olsenella *sp. and *Slackia *sp. The order *Bifidobacteriales *consisted of 398 sequences and 15 phylotypes out of which *Bifidobacterium adolescentis *was the most abundant. Rest of the bifidobacterial OTUs affiliated with *B. catenulatum*, *B. pseudocatenulatum*, *B. bifidum, B. dentium *and *B. longum*. The order *Actinomycetales *comprised of 11 OTUs affiliating with *Actinomyces *sp., *Microbacterium *sp., *Propionibacterium *sp., *Rhodococcus *sp. and *Rothia *sp. (Figure 3).

The unfractioned sample essentially resembled the %G+C fractions 40–45 and 45–50 (Figure 2). In comparison to the combined fractioned clone libraries' the amount of *Firmicutes *(93.2%), especially the percentage of the *Clostridium *cluster XIV (51.0%), increased while the number of *Actinobacteria *(3.5%) decreased. The proportion of *Bacteroidetes *(2.8%) and *Proteobacteria *(0.2%) were the least affected phyla when fractioned and unfractioned libraries were compared (Figure 2, Table 2, Additional file 1). All 16 actinobacterial sequences of the unfractioned library were included in OTUs of the fractioned libraries and *Actinomycetale*s phylotypes were absent in this library (Figure 3). The phyla *Actinobacteria *differed significantly (p = 0.000) between the fractioned and unfractioned libraries in the UniFrac Lineage-specific analysis, though the libraries overall were similar according to the UniFrac Significance test (p = 1.000). Clones from the phylum *Firmicutes *present in the fractioned library but absent in the unfractioned library affiliated with *Enterococcaceae*, *Lactobacillaceae *and *Staphylococcacceae*. Furthermore, only one *Gammaproteobacteria *was found in the unfractioned library whereas the fractioned samples contained also the members of *Alphaproteobacteria*, *Betaproteobacteria *and *Deltaproteobacteria *(Table 2).

### Comparison of individual libraries

The Shared OTUs and Similarity (SONS) program [24] was used to compare the unfractioned sample with each of the %G+C fractions and with the combined sequence data from the fractions (Table 3). Using a 98% similarity criterion for the phylotypes, at least 80% of sequences from %G+C fractions 30–35 and 35–40 were shared with the unfractioned sample (V_obs _values). However, for two of the high %G+C content fractions with %G+C content from 55 to 65, the V_obs _values were considerably lower (32–33%). When comparing the combined sequence data from the fractioned sample with the unfractioned sample, a higher percentage of sequences and OTUs in the unfractioned were shared.

**Table 3:** Results from library comparisons with SONS [24].

Library A	Unfractioned	U_obs_^a^	V_obs_^b^	A_otu_shared_^c^	B_otu_shared_^d^
Library B	Fr G+C 25–30%	0.41	0.40	0.22	0.34

Library B	Fr G+C 30–35%	0.59	0.83	0.40	0.56

Library B	Fr G+C 35–40%	0.67	0.82	0.44	0.64

Library B	Fr G+C 40–45%	0.72	0.75	0.45	0.51

Library B	Fr G+C 45–50%	0.62	0.63	0.33	0.40

Library B	Fr G+C 50–55%	0.34	0.64	0.20	0.40

Library B	Fr G+C 55–60%	0.18	0.33	0.13	0.34

Library B	Fr G+C 60–65%	0.44	0.32	0.17	0.36

Library B	Fr G+C 65–70%	0.68	0.53	0.39	0.39

Library B	Fr G+C 70–75%	0.69	0.67	0.42	0.47

Library B	Fr G+C 25–75%^e^	0.92	0.60	0.81	0.26

a. Fraction of sequences observed in shared OTUs in library A

b. Fraction of sequences observed in shared OTUs in library B

c. Fraction of shared OTUs in library A

d. Fraction of shared OTUs in library B

e. The combined G+C fractions

### Shannon entropies of clone libraries of the %G+C profiled sample

The %G+C fractions 50–55 and 55–60 had comparatively low Shannon entropies (Additional file 2), indicating lower diversity, and were abundant with bifidobacteria (Figure 2, Additional file 1). The peripheral %G+C fractions and the %G+C fraction 45–50 with sequences affiliating mainly with *Clostridium *clusters IV and XIV had comparatively higher diversity according to Shannon entropies. The peripheral fraction from the low %G+C end (25–30% G+C content) contained a substantial proportion of *Firmicutes *that do not belong to the *Clostridum *clusters IV and XIV. It had the highest Shannon entropy (Additional file 2), indicating rich diversity, and did not reach a plateau in the rarefaction curves (data not shown), which means that more OTUs would have been likely to appear after further sequencing.

## Discussion

For a comprehensive evaluation of the human intestinal microbiota, 16S rRNA gene clone libraries were constructed from a %G+C fractioned pooled faecal DNA sample of 23 healthy subjects followed by a sequence analysis of 3199 clones. Previously, only selected fractions of such profiles have been sequenced and analysed. For methodological comparison, a 16S rRNA gene library of unfractioned DNA from 22 individuals representing the same subject group was also constructed. The %G+C fractioning prior to cloning and sequencing enhanced the recovery of sequences affiliating with high G+C Gram-positive bacteria, namely the phylum *Actinobacteria*, proportionally over sevenfold compared with cloning and sequencing of an unfractioned sample.

### A high amount of actinobacterial sequences recovered

If the proportional amount of DNA in each fraction is taken into account in estimating the abundance of phyla, 28.5% of the sequences would affiliate with *Actinobacteria*. Since the %G+C profile fractions represent individual cloning and sequencing experiments, in which an equal amount of clones were sequenced despite the different proportional amounts of DNA within the fractions, quantitative conclusions should be drawn carefully. However, %G+C fractions 50–70 were dominated by *Actinobacteria*, comprising 41% of the total DNA in the original sample fractioned (Figures 1 and 2, Additional file 1). The %G+C fractions 30–50 yield a similar phylotype distribution as the unfractioned library (Figure 2). These fractions, accounting for 54% of the profiled DNA, are dominated by the *Firmicutes *(*Clostridium *clusters XIV and IV) (Figure 1 and 2).

The relatively high proportion of actinobacterial sequences (26.6%) and phylotypes (65) identified in the combined sequence data of the %G+C fractioned sample exceed all previous estimations. In a metagenomic study by Gill and colleagues [14], 20.5% of 132 16S rRNA sequences from random shotgun assemblies affiliated with 10 phylotypes of *Actinobacteria *whereas no *Bacteroidetes *was detected. In accordance with our results, also a pyrosequencing study by Andersson and colleagues [16], the *Actinobacteria *(14.6%), dominated by a few phylotypes, outnumbered *Bacteroidetes *(2.5%). By contrast, in most of the earlier published studies on human faecal samples applying 16S rRNA gene amplification, cloning and sequencing, the relative amount of *Actinobacteria *has been 0–6% of the detected intestinal microbiota [12,25-33]. Thus, the proportion of sequences affiliating with *Actinobacteria *(3.5%) in the unfractioned sample analysed in this study is comparable with previous estimations applying conventional 16S rRNA cloning and sequencing without %G+C fractioning.

### Order Coriobacteriales abundant within Actinobacteria

We observed that several clones in the high %G+C fractions (60–70% G+C content) were tricky to sequence due to extremely G+C rich regions. These clones turned out to be members of order *Coriobacteriales*, which have been rare or absent in earlier 16S rRNA gene -based clone libraries of the intestinal microbiota. Over half of the actinobacterial OTUs in our study belonged to the order *Coriobacteriales*. Harmsen *et al*. [34] earlier suggested that applications based on 16S rRNA gene cloning as well as other methods of molecular biology may overlook the presence of the family *Coriobacteriaceae *in the human GI tract and they designed a group-specific probe for *Atopobium *(Ato291), covering most of the *Coriobacteriaceae*, the *Coriobacterium *group. Using Ato291, the abundance of detected intestinal cells in fluorescence *in situ *hybridization (FISH) is up to 6.3%. [6,7,35,36]. Recently, Khachatryan and colleagues [8] did not detect any *Actinobacteria *from the 16S rRNA gene clone libraries of healthy subjects but the abundance with FISH using Ato291 was 7%. The authors suggested that constant underestimation of the high G+C Gram-positive bacteria might lead to misunderstanding their role in the healthy and diseased gut.

There are some data suggesting that the members of *Coriobacteriaceae *may be indicators of a healthy GI microbiota. Subjects with a low risk of colon cancer have been observed to have a higher incidence of *Collinsella aerofaciens *than subjects with a high risk of colon cancer [37]. Furthermore, when faecal 16S rRNA gene sequences from metagenomic libraries of Crohn's diseased and healthy subjects were compared, the *Atopobium *group was more prevalent and the groups designated "other *Actinobacteria*" were exclusively detected in healthy subjects' samples [11]. A lower abundance of a *C. aerofaciens*-like phylotype within the *Atopobium *group has been associated with IBS subjects' samples [21]. Diminished amount of *Atopobium *group bacteria is also associated with patients with Mediterranean fever [8]. On the other hand, increased amount of *Actinobacteria *have recently been associated with the faecal microbiota of obese subjects [32]. This indicates that more detailed data are required to judge the role of *Actinobacteria *in health and disease.

### Methodological observations

When the %G+C gradient is disassembled, the fractions with the highest G+C content are collected last, making them most susceptible to turbulence. This phenomenon together with possible remnants of DNA from previously collected fractions could have caused the bias of a decrease in high G+C *Actinobacteria *and an increase in low G+C *Firmicutes *observed in fractions %G+C 65–75. These fractions, however, comprise only 5.5% of the total DNA, making the observed bias less important. Regarding faecal DNA extraction, the method used here was rather rigorous, allowing efficient DNA isolation also from more enduring Gram-positive bacteria. This might lower the relative amount of DNA from more easily lysed Gram-negative bacteria and thus explain the comparatively low amount of *Bacteroides *in both of the samples. Moreover, the relative share of *Bacteroidetes *phyla may be affected by the delay and temperature of freezing. In a real-time PCR study, a decrease of 50% in the *Bacteroides *group was observed in faecal sample aliquots frozen in -70°C within 4 h compared to samples that were immediately snap-frozen in liquid nitrogen (Salonen *et al*., personal communication). In our study, the samples were transported within 4 h of the defecation and stored at -70°C.

Abundance of *Actinobacteria *in the faeces of Scandinavian (Finnish and Swedish) subjects has been discovered independent of the methodology; the techniques used include %G+C profiling and 16S rDNA gene cloning (this study), FISH coupled with flow cytometry [7] and pyrosequencing [16]. These findings may suggest existence of demographic similarities among Scandinavians, which could be caused by environmental or genetic factors and that are not obscured by methodological bias of DNA extraction, primers and PCR conditions used.

## Conclusion

The results further confirm that %G+C fractioning is an efficient method prior to PCR amplification, cloning and sequencing to obtain a more detailed understanding of the diversity of complex microbial communities, especially within the high genomic %G+C content region. This is proven by the proportionally greater amount of OTUs and sequences affiliating with the high G+C Gram-positive phylum *Actinobacteria *in the 16S rRNA gene clone libraries originating from a %G+C-profiled and -fractioned faecal microbial genomic DNA sample compared with a sample cloned and sequenced without prior %G+C profiling. The clone content obtained from the unfractioned library is in accordance with many previous clone library analyses and thus suggests that the potential underestimation of high G+C gram positive bacteria, have hidden the importance of these bacteria in a healthy gut. The phyla *Actinobacteria *were the second most abundant phyla detected in the %G+C fractioned sample consisting mainly of sequences affiliating with mainly *Coriobacteriaceae*.

## Methods

### Study subjects

The faecal samples were collected from 23 healthy donors (females n = 16, males n = 7), with an average age of 45 (range 26–64) years, who served as controls for IBS studies [21,38-40]. Exclusion criteria for study subjects were pregnancy, lactation, organic GI disease, severe systematic disease, major or complicated abdominal surgery, severe endometriosis, dementia, regular GI symptoms, antimicrobial therapy during the last two months, lactose intolerance and celiac disease. All participants gave their written informed consent and were permitted to withdraw from the study at any time.

### Faecal DNA samples

Faecal samples were immediately stored in anaerobic conditions after defecation, aliquoted after homogenization and stored within 4 h of delivery at -70°C. The bacterial genomic DNA from 1 g of faecal material was isolated according to the protocol of Apajalahti and colleagues [41]. Briefly, undigested particles were removed from the faecal material by three rounds of low-speed centrifugation and bacterial cells were collected with high-speed centrifugation. The samples were then subjected to five freeze-thaw cycles, and the bacterial cells were lysed by enzymatic (lysozyme and proteinase K) and mechanical (vortexing with glass beads) means. Following cell lysis, the DNA was extracted and precipitated.

### Percent guanine plus cytosine fractioning and purification of fractions

The faecal microbial DNA of 23 healthy individuals was pooled, and genomic DNA fractions were separated with 5% intervals on the basis of %G+C content using caesium chloride-bisbenzimidazole gradient analysis described in previous studies [21,41]. The gradient was disassembled into %G+C fractions with 5 G+C% intervals using perfluorocarbon (fluorinert) as a piston. In the procedure, the highest %G+C fraction is collected last, exposing it to the most turbulence. The DNA quantification during the dismantlement was based on A_280_, as described by Apajalahtiand colleagues [41], to avoid background. The DNA fractions were desalted with PD-10 columns according to the manufacturer's instructions (Amersham Biosciences, Uppsala, Sweden). For the unfractioned DNA sample, faecal microbial DNA of the same healthy individuals was pooled (n = 22; there was an insufficient amount of faecal DNA left for one of the individuals).

### Amplification of the 16S rRNA genes, cloning and sequencing

The 16S rRNA gene from each of the seven DNA fractions was amplified, cloned and sequenced, as in the study by Kassinen and colleagues [21]. To maximize the recovery of different phylotypes, two universal primer pairs were used independently for all samples. The first primer pair corresponded to *Escherichia coli *16S rRNA gene positions 8–27 and 1492–1512, with sequences 5'-AGAGTTTGATCCTGGCTCAG-3' [42] and 5'-ACGGCTACCTTGTTACGACTT-3' [43], respectively. The second primer pair corresponded to *E. coli *16S rRNA gene positions 7–27 and 1522–1541, with sequences 5'-GAGAGTTTGATYCTGGCTCAG-3' and 5'-AAGGAGGTGATCCARCCGCA-3' [44], respectively. The 50-μl PCR reactions contained 1 × DyNAzyme™ Buffer (Finnzymes, Espoo, Finland), 0.2 mM of each dNTP, 50 pmol of primers, 1 U of DyNAzyme™ II DNA Polymerase (Finnzymes, Espoo, Finland), 0.125 U of Pfu DNA polymerase (Fermentas, Vilnius, Lithuania) and 10 μl of desalted fractioned DNA template (containing less than 2 ng/μl of DNA) or pooled extracted DNA from the faecal samples. The thermocycling conditions consisted of 3 min at 95°C, followed by a variable number of cycles of 30 s at 95°C, 30 s at 50°C, 2 min at 72°C and a final extension of 10 min at 72°C. The number of PCR cycles used for each fraction was optimized to the minimum amount of cycles which resulted in a visually detectable band of the PCR product on ethidium bromide stained agarose gel. A protocol of 27, 20, 25 and 30 cycles was applied to %G+C fraction 25–30, 30–60, 60–65 and 65–75, respectively. The 16S rRNA gene from the unfractioned pooled faecal DNA sample was amplified using 20 PCR cycles. The amplifications were performed using 15 reactions, and the products were pooled, concentrated using ethanol precipitation, and eluted with 50 μl of deionized MilliQ water (Millipore, Billerica, MA, USA).

The precipitated PCR products were purified with the QIAquick PCR Purification Kit (Qiagen, Hilden, Germany), or using the QIAquick Gel Extraction Kit (Qiagen, Hilden, Germany) after excising from 1.25% SeaPlaque agar (Cambrex, East Rutherford, NJ, USA), and eluted in 35 μl of elution buffer. The concentration of the purified amplicons was estimated with serially diluted samples on 0.8% agarose gels with ethidium bromide staining. To enhance the cloning efficiency, adenine overhangs were added to the amplicons as follows: The two purified inserts were mixed in a 1:1 molecular ratio (the reaction mixture thus contained 10–30 ng/μl DNA) and incubated in a volume of 20 μl with 1 × DyNAzyme™ Buffer (Finnzymes, Espoo, Finland), 0.2 mM dNTPs and 0.4 U of DyNAzyme™ II DNA Polymerase (Finnzymes, Espoo, Finland) for 40 min at 72°C. The cloning was performed with the QIAGEN^® ^PCR Cloning plus Kit (Qiagen, Hilden, Germany) according to the manufacturer's instructions. For the ligation reaction, 2 μl of the reaction mixture used for adding adenine overhangs to the amplicons was used as an insert. The ligation reaction was incubated overnight at 4°C. The plasmids were isolated and purified from the *E. coli *culture using MultiScreen_HTS _(Millipore, Billerica, MA, USA), and aliquots were stored in -80°C. 

The cloned inserts were amplified from the pDrive plasmids using M13 forward 5'-GTAAAACGACGGCCAGT-3' and M13 reverse primers 5'-AACAGCTATGACCATG-3', visualized on a 1% agarose gel, stained with ethidium bromide and purified using a MultiScreen PCR_384 _Filter Plate (Millipore, Billerica, MA, USA). Sequencing of the 5'-end of 16S rDNA clones was performed with primer pD' 5'-GTATTACCGCGGCTGCTG-3' corresponding to the *E. coli *16S rRNA gene position 536-518 [45]. Near full-length sequencing was performed on one representative of each OTU showing less than 95% similarity to any EMBL nucleotide sequence database entry. For this purpose, primers pF' 5'-ACGAGCTGACGACAGCCATG-3' [45] and pE 5'-AAACTCAAAGGAATTGACGG-3' [46], corresponding to *E. coli *16S rRNA gene positions 1073-1053 and 908–928, respectively, were used. Sequencing of the products was performed with the BigDye terminator cycle sequencing kit (Applied Biosystems, Foster City, CA, USA). For templates that failed to be sequenced due to high G+C content, 1% (v/v) of dimethyl sulfoxide was added to the reaction mixture. The sequencing products were cleaned with Montage SEQ_96 _plates (Millipore, Billerica, MA, USA) and run with an ABI 3700 Capillary DNA Sequencer (Applied Biosystems, Foster City, CA, USA).

### Sequence analysis and alignment

Sequences were checked manually utilizing the Staden Package pregap4 version 1.5 and gap v4.10 assembly programs [47], and primer sequences were removed. Sequences that occurred in more than one clone library were considered non-chimeric. Revealing the potential chimeras was also performed by manually browsing the ClustalW 1.83 sequence alignment [48] with Bio Edit version 7.0.5.3 [49] and for the near full-length sequences using Ribosomal Database Project II Chimera Check [50]. Sequences from %G+C fractions 25–30, 40–45 and 55–60 with accession numbers AM275396-AM276371 [21] were added prior to further analyses. Sequences of all fractions and the unfractioned sample were aligned separately with ClustalW 1.83 [48] using the FAST DNA pair-wise alignment algorithm option (Gap penalty 3, Word size 4, Number of top diagonals 1 and Window size 1) and cut from *E. coli *position 430 (totally conserved GTAAA) with BioEdit version 7.0.5.3 [49]. The lengths of the alignments of the fractioned sample and the unfractioned sample were 478 and 457 base pairs, respectively. The 16S rRNA variable regions V1 and V2 were included in the alignments. The variable regions V1 and V2 have been demonstrated to be sufficient to reflect the diversity of a human GI clone library [51]. The alignments were visually inspected, but they were not edited manually to avoid subjectivity and to maintain reproducibility of the alignments. From the cut alignments, distance matrices were created with Phylip 3.66 Dnadist [52] using Jukes-Cantor correction.

### Determination of OTUs and library coverage

The sequences were assigned into OTUs according to the distance matrices using DOTUR [53], applying the furthest neighbour rule option in which all sequences within an OTU fulfil the similarity criterion with all the other sequences within the OTU. The 98% cut-off for sequence similarity was used to delimit an OTU. The coverage of the clone libraries was calculated with the formula of Good [23] to evaluate the adequacy of amount of sequencing. The Fasta EMBL Environmental and EMBL Prokaryote database searches [54] and Ribosomal Database Project II (RDP II) Classifier Tool [55] were used to affiliate phylotypes.

### Phylogenetic analysis

For the phylogenetic analysis, all sequences from the %G+C fractioned sample and the unfractioned sample were aligned and designated into OTUs with a 98% cut-off as described above. A representative sequence of each OTU and unaligned reference sequences representing different clostridial groups (Additional file 3) were aligned with ClustalW 1.83 using the SLOW DNA alignment algorithm option (Gap penalty 3, Word size 1, Number of top diagonals 5 and Window size 5) and cut from the *E. coli *position 430 (totally conserved GTAAA) with BioEdit version 7.0.5.3[49]. For a profile alignment, 16S rRNA reference sequences, aligned according to their secondary structure, were selected from the European ribosomal RNA database [56] (Additional file 4) so that they would represent the overall diversity of the faecal microbiota, including the most common clostridial 16S rRNA groups expected, and sequences closely related to the OTUs composed of over 20 sequences. The sequences in this study were profile-aligned against the European ribosomal RNA database secondary structure-aligned sequences using ClustalW 1.83 profile alignment mode and the SLOW DNA alignment algorithm option (Gap penalty 3, Word size 1, Number of top diagonals 5 and Window size 5). The reference sequences were then deleted from the alignment with BioEdit version 7.0.5.3 [49], and the alignment was cut at the *E. coli *position 430 (totally conserved GTAAA). A phylogenetic tree with a representative sequence from each OTU was generated with a neighbour-joining algorithm from a Jukes-Cantor-corrected distance matrix using Phylip 3.66 dnadist and neighbour [52]. The tree was visualized with MEGA4 [57].

A phylogenetic tree was constructed for the OTU representatives of the phylum *Actinobacteria*. For *Bifidobacteriales *and *Actinomycetales*, sequences with nearest FASTA EMBL Prokaryote search (all >98% similarity), and for *Coriobacteriales *sequences with nearest FASTA EMBL prokaryote and environmental database searches (>85% and >91%, respectively), were selected and aligned together with OTU representative sequences. Sequences from the European ribosomal RNA database representing *Actinobacteria *and *Clostridium leptum *(AF262239) were used as a reference in the profile alignment (Additional file 4). The alignment, distance matrix, and visualizing was done as described above. A bootstrap analysis of hundred replicates was performed using seqboot and consense programs of Phylip 3.66 [52].

To describe whether the phylogenies of the combined sequence data from the fractioned libraries and the unfractioned library were significantly different, the UniFrac Significance analysis was applied for each pair of environments using abundance weights [58]. The UniFrac Lineage-specific analysis was used to break the tree up into the lineages at a specified distance from the root, and to test whether any particular group differed between the sample libraries [58]. The phylogenetic tree for the analyses was constructed from OTU representative sequences determined separately for the combined fractioned libraries and for the unfractioned library as described above, with the exception that in the profile alignment a root sequence (*Methanobrevibacter smithii *AF054208) was added and left to the alignment.

### Comparison of individual libraries using SONS

The microbial community composition differences between libraries of individual %G+C profile fractions and the unfractioned sample were analysed using SONS [24], which calculates the fraction of sequences observed in shared OTUs in each library (U_obs _and V_obs_) and the observed fraction of shared OTUs in each library (A_otu_shared _and B_otu_shared_). For the SONS analyses, an alignment with all of the sequences from the clone libraries of the fractioned sample and the unfractioned sample was created, and a distance matrix was calculated as described above in the *Sequence analysis and alignment *section.

### Shannon entropies of clone libraries of the %G+C profiled sample

To compare the diversity of the clone libraries derived from the fractioned sample, OTUs were also determined using a Bayesian clustering method [59], followed by the estimation of Shannon entropies with a standard Bayesian multinomial-Dirichlet model. In the estimation, 100 000 Monte Carlo samples were used for each library under a uniform Dirichlet prior [60]. The Shannon entropy value correlates with the amount and evenness of clusters or phylotypes in a community sample, but disregards the disparity between them [61]. The Bayesian clustering method groups the sequences into clusters more distinct from each other than would, for example, the ClustalW alignment-based Jukes-Cantor-corrected distance matrices, demanding more disparity among the sequences present in a sample for them to form separate clusters.

### Nucleotide sequence accession numbers

The 16S rRNA gene sequences reported in this study have been deposited in the EMBL Nucleotide Sequence Database under accession numbers AM404446–AM406668 and AM888398–AM888856.

## Authors' contributions

LK-K and AK conducted the sequence analysis and prepared the manuscript, LP supervised the sequencing library construction procedure, JC determined the Shannon entropies, HM performed %G+C fractioning of the pooled DNA samples, JT acted as bioinformatics specialist and provided scripts needed in data analysis for LK-K and AK, AP designed and supervised the study. All authors have contributed in the manuscript writing process as well as approved the final manuscript.

## Supplementary Material

Additional File 1 **Affiliation of OTUs derived from the %G+C fractioned sample**. Classification of OTUs to phyla utilizing RDB Classifier [55], nearest similarity to EMBL prokaryote database sequences [54] and the number of sequences in individual %G+C fractions. Click here for file

Additional File 2 **Comparison of the %G+C clone library diversities using Shannon entropy**. The Shannon entropy values correlate with the amount and evenness of clusters or phylotypes in a community sample, but disregard the disparity between them. Click here for file

Additional File 3 ***Clostridium *cluster reference sequences**. Unaligned *Clostridium *cluster reference sequences used in the phylogenetic analysis of sequence data. Click here for file

Additional File 4 **Reference sequences from the European ribosomal RNA database **[56]. Reference sequences aligned according to their secondary structure and used in the phylogenetic analysis of sequence data. Click here for file
