# Supplementary material for: Sequence analysis of percent G+C fraction libraries of human faecal bacterial DNA reveals a high number of Actinobacteria
Source: BMC Microbiol. 2009 Apr 8;9:68. doi: 10.1186/1471-2180-9-68 (PMC2679024; doi:10.1186/1471-2180-9-68)
Supplement: Additional File 1 — Affiliation of OTUs derived from the %G+C fractioned sample. Classification of OTUs to phyla utilizing RDB Classifier [55], nearest similarity to EMBL prokaryote database sequences [54] and the number of sequences in individual %G+C fractions. [file 1471-2180-9-68-S1.pdf]

Additional file 1 - Affiliation of OTUs derived from the %G+C fractioned sample.

*Novel OTUs with less than 95% sequence similarity to public sequence database (in November 2006)

Representative sequence	Sequenced to near full-length*	RDP II Classifier Phylum	Similarity (%)	EMBL Prokaryote database Closest match	Description	Identity (%)	Sum of sequences in all fractions	Sum of sequences in individual fractions (G+C content of fractions) 25-30%	30-35%	35-40%	40-45%	45-50%	50-55%	55-60%	60-65%	65-70%	70-75%
AM406398		Acidobacteria	100	AF498753	Bacterium Ellin371	94,4	1	0	0	0	0	0	0	0	0	0	1
AM275496		Actinobacteria	100	AB064927	Bifidobacterium sp. CS16	99,8	43	1	0	0	1	3	11	21	1	2	3
AM275529		Actinobacteria	100	AE014295	Bifidobacterium longum NCC2705	100	82	1	0	0	1	1	10	37	14	12	6
AM275737		Actinobacteria	75	AF079506	Denitrobacterium detoxifican strain NPOH3	85,6	1	0	0	0	1	0	0	0	0	0	0
AM275753		Actinobacteria	100	AJ131149	Coriobacterium sp.	98,7	26	0	0	0	1	0	0	20	3	2	0
AM275770		Actinobacteria	100	AY305304	Bifidobacterium adolescentis isolate L2-32	100	73	0	0	0	1	3	11	20	10	23	5
AM275775		Actinobacteria	31	AF079506	Denitrobacterium detoxifican strain NPOH3	84,8	1	0	0	0	1	0	0	0	0	0	0
AM276066		Actinobacteria	100	AY937380	Eggerthella lenta strain SECO-Mt75m2	99,8	7	0	0	0	0	0	0	1	4	2	0
AM276076		Actinobacteria	98	AF101241	Slackia heliotrinreducens	89,6	1	0	0	0	0	0	0	1	0	0	0
AM276078		Actinobacteria	100	AF292375	Eggerthella lenta	100	8	0	0	0	0	0	0	1	7	0	0
AM276079		Actinobacteria	100	U25951	Bifidobacterium bifidum KCTC 3202	99,3	17	0	0	0	0	0	0	5	8	3	1
AM276080		Actinobacteria	100	AY305304	Bifidobacterium adolescentis isolate L2-32	98,3	2	0	0	0	0	0	0	1	0	1	0
AM276083		Actinobacteria	100	AB064927	Bifidobacterium sp. CS16	98,3	4	0	0	0	0	0	0	2	2	0	0
AM276086	X	Actinobacteria	89	AJ608686	Slackia faecicanis, type strain CCUG 48399	91,0	11	0	0	0	0	0	0	3	6	2	0
AM276088		Actinobacteria	100	D86183	Bifidobacterium dentium, strain: ATCC 27534	99,1	1	0	0	0	0	0	0	1	0	0	0
AM276097		Actinobacteria	100	AY310748	Human intestinal bacterium SNU-Julong732	92,6	2	0	0	0	0	0	0	1	1	0	0
AM276104		Actinobacteria	99	AY310748	Human intestinal bacterium SNU-Julong732	99,8	22	0	0	0	0	0	0	5	10	6	1
AM276105		Actinobacteria	98	AY310748	Human intestinal bacterium SNU-Julong732	97,8	44	0	0	0	0	0	0	4	30	8	2
AM276106		Actinobacteria	100	AJ131149	Coriobacterium sp.	98,2	62	0	0	0	0	0	0	35	15	8	4
AM276108		Actinobacteria	96	AF292375	Eggerthella lenta	90,6	14	0	0	0	0	0	0	5	8	1	0
AM276111		Actinobacteria	100	AB116272	Bifidobacterium adolescentis, strain: JCM 7045	99,3	12	0	0	0	0	0	0	5	4	2	1
AM276117		Actinobacteria	100	AB064935	Collinsella sp. CB20	98,9	28	0	0	0	0	0	0	17	8	3	0
AM276190		Actinobacteria	100	AB064935	Collinsella sp. CB20	99,1	3	0	0	0	0	0	0	2	1	0	0
AM404803	X	Actinobacteria	63	AY998134	Bacterium ROMEm59sa331	83,9	2	0	0	1	0	0	0	0	0	0	1
AM404821		Actinobacteria	100	AF292375	Eggerthella lenta	93,3	38	0	0	1	2	7	15	5	3	4	1
AM404828		Actinobacteria	53	AY998134	Bacterium ROMEm59sa331	83,6	2	0	0	1	0	0	0	0	0	1	0
AM405132	X	Actinobacteria	63	AF325325	Atopobium vaginae	86,2	1	0	0	0	0	1	0	0	0	0	0
AM405134		Actinobacteria	100	AB037383	Collinsella sp. RCA56-80	98,6	33	0	0	0	0	2	0	2	27	2	0
AM405160		Actinobacteria	100	AF432082	Bifidobacterium catenulatum	100	45	0	0	0	0	3	12	6	11	7	6
AM405200		Actinobacteria	100	AY856700	Bifidobacterium sp. H12	98,4	2	0	0	0	0	1	0	0	1	0	0
AM405205		Actinobacteria	95	AF101241	Slackia heliotrinreducens	88,7	4	0	0	0	0	1	0	0	2	1	0
AM405218		Actinobacteria	100	AJ131150	Coriobacterium sp.	100	75	0	0	0	0	4	0	40	15	13	3
AM405234		Actinobacteria	100	AB116273	Bifidobacterium adolescentis	98,1	42	0	0	0	0	2	10	9	15	4	2
AM405434		Actinobacteria	100	AF275881	Bifidobacterium adolescentis clone nru-1	98,1	28	0	0	0	0	0	5	9	10	4	0
AM405450		Actinobacteria	100	AJ234042	Actinomyces odontolyticus	98,4	3	0	0	0	0	0	1	1	0	1	0
AM405455		Actinobacteria	100	AF275881	Bifidobacterium adolescentis clone nru-1	99,5	2	0	0	0	0	0	2	0	0	0	0
AM405458		Actinobacteria	100	AB064927	Bifidobacterium sp. CS16	98,4	14	0	0	0	0	0	5	4	3	1	1
AM405465		Actinobacteria	100	AJ608686	Slackia faecicanis, type strain CCUG 48399	92,9	17	0	0	0	0	0	5	4	3	3	2
AM405487	X	Actinobacteria	52	AY321959	Eggerthella hongkongensis strain HKU11	86,9	3	0	0	0	0	0	1	0	2	0	0
AM405495		Actinobacteria	100	D86187	Bifidobacterium pseudocatenulatum	99,8	7	0	0	0	0	0	5	2	0	0	0
AM405498		Actinobacteria	100	AF306789	Bifidobacterium sp. PL1	100	17	0	0	0	0	0	4	5	5	3	0
AM405508		Actinobacteria	100	AF306789	Bifidobacterium sp. PL1	98,1	7	0	0	0	0	0	4	2	1	0	0
AM405720	X	Actinobacteria	98	AJ518870	Denitrobacterium sp. CCUG 45655	94,9	1	0	0	0	0	0	0	0	1	0	0
AM405723	X	Actinobacteria	100	AF292375	Eggerthella lenta	90,4	1	0	0	0	0	0	0	0	1	0	0
AM405728	X	Actinobacteria	48	AY937380	Eggerthella lenta strain SECO-Mt75m2	89,1	1	0	0	0	0	0	0	0	1	0	0
AM405747		Actinobacteria	100	Y10819	P. freudenreichii subsp. shermanii	100	2	0	0	0	0	0	0	0	2	0	0
AM405748	X	Actinobacteria	99	AY239459	Eubacterium sp. cL-10-1-3	91,0	1	0	0	0	0	0	0	0	1	0	0
AM405783		Actinobacteria	100	AY321958	Eggerthella sinensis strain HKU14	92,2	2	0	0	0	0	0	0	0	2	0	0
AM405784	X	Actinobacteria	100	AF292373	Olsenella uli	95,4	1	0	0	0	0	0	0	0	1	0	0
AM405787		Actinobacteria	100	AY288517	Eggerthella hongkongensis	91,0	5	0	0	0	0	0	0	0	3	2	0
AM405897		Actinobacteria	100	AF101241	Slackia heliotrinreducens	87,9	4	0	0	0	0	0	0	0	4	0	0
AM406030	X	Actinobacteria	98	AF101241	Slackia heliotrinreducens	87,5	1	0	0	0	0	0	0	0	0	1	0
AM406041		Actinobacteria	100	AY504471	Microbacteriaceae bacterium C53	99,8	1	0	0	0	0	0	0	0	0	1	0
AM406043		Actinobacteria	100	AF292375	Eggerthella lenta	92,6	1	0	0	0	0	0	0	0	0	1	0
AM406048		Actinobacteria	100	AY278611	Actinomyces genomosp. C2	97,7	1	0	0	0	0	0	0	0	0	1	0
AM406058		Actinobacteria	100	AJ234052	Actinomyces naeslundii,strain CCUG 34286, clone 2	97,4	1	0	0	0	0	0	0	0	0	1	0
AM406063		Actinobacteria	100	AM181504	Microbacterium hominis, type strain DSM 12509	98,8	2	0	0	0	0	0	0	0	0	1	1
AM406065	X	Actinobacteria	86	AY239459	Eubacterium sp. cL-10-1-3	92,5	1	0	0	0	0	0	0	0	0	1	0
AM406090	X	Actinobacteria	99	AY321960	Eggerthella hongkongensis strain HKU12	89,3	1	0	0	0	0	0	0	0	0	1	0
AM406113		Actinobacteria	100	AJ518870	Denitrobacterium sp. CCUG 45655	98,7	2	0	0	0	0	0	0	0	0	2	0
AM406149		Actinobacteria	100	AJ234055	Actinomyces naeslundii, strain CCUG 34808	98,5	2	0	0	0	0	0	0	0	0	2	0
AM406157		Actinobacteria	100	DQ185597	Rhodococcus sp. djl-6-2	100	10	0	0	0	0	0	0	0	0	3	7
AM406435		Actinobacteria	100	DQ409140	Rothia mucilaginosa	99,5	1	0	0	0	0	0	0	0	0	0	1
AM406440	X	Actinobacteria	100	AJ245921	Coriobacterium sp. EKSO3	95,6	1	0	0	0	0	0	0	0	0	0	1
AM406441		Actinobacteria	100	AE017283	Propionibacterium acnes KPA171202	100	1	0	0	0	0	0	0	0	0	0	1
AM406445	X	Actinobacteria	100	AF292375	Eggerthella lenta	93,9	1	0	0	0	0	0	0	0	0	0	1
AM275402		Bacteroidetes	100	AE015928	Bacteroides thetaiotaomicron	100	4	2	1	1	0	0	0	0	0	0	0
AM275453		Bacteroidetes	100	AB214328	Bacteroides intestinalis	98,9	1	1	0	0	0	0	0	0	0	0	0
AM275521		Bacteroidetes	99	AE015928	Bacteroides thetaiotaomicron	91,1	1	1	0	0	0	0	0	0	0	0	0
AM275607		Bacteroidetes	100	AY643080	Bacteroides sp. Smarlab 3301643	100	2	1	0	0	0	0	0	0	0	1	0
AM275618		Bacteroidetes	95	AJ581354	Prevotella sp. BI-42	98,5	2	1	0	0	0	0	0	0	1	0	0
AM275634		Bacteroidetes	100	AF357553	Bacterium mpn-isolate	100	12	1	2	6	1	2	0	0	0	0	0
AM275642		Bacteroidetes	100	AY126616	Bacteroides massiliensis B84634	99,8	1	1	0	0	0	0	0	0	0	0	0
AM275718		Bacteroidetes	99	AB239491	Rumen bacterium RC-2	88,7	2	0	0	0	1	1	0	0	0	0	0
AM275723		Bacteroidetes	91	AF218617	Prevotella ruminicola strain TC2-3	88,6	2	0	0	0	1	0	0	0	0	1	0
AM275747		Bacteroidetes	68	AB239491	Rumen bacterium RC-2	82,7	3	0	0	0	1	2	0	0	0	0	0
AM275765		Bacteroidetes	90	AY239469	Gram-negative bacterium cL10-2b-4	84,6	2	0	0	0	1	0	1	0	0	0	0
AM275769		Bacteroidetes	100	AJ518872	Bacteroides sp. CCUG 39913	100	1	0	0	0	1	0	0	0	0	0	0
AM275776		Bacteroidetes	100	AB075682	Bacteroides sp. C35	96,6	1	0	0	0	1	0	0	0	0	0	0
AM275777		Bacteroidetes	100	AB064914	Bacteroides sp. CS27	99,2	1	0	0	0	1	0	0	0	0	0	0
AM275800		Bacteroidetes	100	AB238928	Parabacteroides merdae	98,4	2	0	0	0	2	0	0	0	0	0	0
AM404450		Bacteroidetes	87	AB239491	Rumen bacterium RC-2	89,5	9	0	1	2	6	0	0	0	0	0	0
AM404483		Bacteroidetes	100	DQ100446	Bacteroides ovatus strain SDG-Mt85-3Cy	98,7	3	0	1	0	0	0	0	0	0	2	0
AM404504		Bacteroidetes	100	AF139524	Bacteroides sp. AR20	100	16	0	1	4	7	2	0	0	0	1	1
AM404521		Bacteroidetes	99	AY608697	Bacteroides sp. WAL 11050	92,6	3	0	1	0	1	0	0	0	0	1	0
AM404531		Bacteroidetes	100	AB200218	Bacteroides plebeius	99,3	2	0	1	0	0	0	0	0	0	1	0
AM404547		Bacteroidetes	100	AB242142	Bacteroides sp. 175T	100	5	0	4	0	0	0	0	0	0	1	0
AM404800		Bacteroidetes	100	DQ100446	Bacteroides ovatus strain SDG-Mt85-3Cy	95,5	1	0	0	1	0	0	0	0	0	0	0
AM404813		Bacteroidetes	100	DQ100446	Bacteroides ovatus strain SDG-Mt85-3Cy	97,3	1	0	0	1	0	0	0	0	0	0	0
AM404832		Bacteroidetes	100	AB064911	Bacteroides sp. CS24	99,8	3	0	0	1	1	0	0	0	0	1	0
AM404866		Bacteroidetes	100	AB080888	Bacteroides sp. CJ89	99,1	1	0	0	1	0	0	0	0	0	0	0
AM404874		Bacteroidetes	100	L16497	Bacteroides putredinis ATCC 29800	98,0	3	0	0	2	1	0	0	0	0	0	0
AM405133		Bacteroidetes	100	AY643083	Alistipes finegoldii CIP 107999	99,6	2	0	0	0	0	1	0	0	0	1	0
AM405151		Bacteroidetes	57	AY862593	Bacterium L4M2 1-9	85,0	2	0	0	0	0	1	0	0	1	0	0
AM405191		Bacteroidetes	100	L16497	Bacteroides putredinis ATCC 29800	90,2	1	0	0	0	0	1	0	0	0	0	0
AM405220		Bacteroidetes	89	AF357576	Bacterium mpn-isolate group 3	98,6	1	0	0	0	0	1	0	0	0	0	0
AM405230	X	Bacteroidetes	94	AB239491	Rumen bacterium RC-2	89,0	1	0	0	0	0	1	0	0	0	0	0
AM405432		Bacteroidetes	97	AY974071	Alistipes sp. WAL 8169	87,8	1	0	0	0	0	0	1	0	0	0	0
AM405473		Bacteroidetes	87	AY239469	Gram-negative bacterium cL10-2b-4	85,4	2	0	0	0	0	0	2	0	0	0	0
AM405752		Bacteroidetes	97	AY974072	Alistipes sp. WAL 8301	87,2	1	0	0	0	0	0	0	0	1	0	0
AM406411		Bacteroidetes	97	AB200225	Bacteroides coprocola	94,2	1	0	0	0	0	0	0	0	0	0	1
AM406416		Bacteroidetes	100	AY974072	Alistipes sp. WAL 8301	99,6	1	0	0	0	0	0	0	0	0	0	1
AM406500		Bacteroidetes	100	AJ252601	Rhizosphere soil bacterium isolate RSI-34	90,0	2	0	0	0	0	0	0	0	0	0	2
AM406444		Cyanobacteria	100	CP000110	Synechococcus sp. CC9605	86,2	2	0	0	0	0	0	0	0	0	0	2
AM275396		Firmicutes	89	AY949859	Clostridium sp. strain Z6	89,5	2	1	1	0	0	0	0	0	0	0	0
AM275397		Firmicutes	69	AJ270479	Butyrate-producing bacterium	86,1	8	8	0	0	0	0	0	0	0	0	0
AM275398		Firmicutes	69	AB239485	Rumen bacterium R-15	82,9	14	10	3	1	0	0	0	0	0	0	0
AM275399		Firmicutes	100	DQ445858	Clostridium sp. C01-2409	97,6	17	14	0	1	0	0	0	0	0	1	1
AM275400		Firmicutes	100	AB064890	Human intestinal firmicute CB17	97,4	7	1	5	0	1	0	0	0	0	0	0
AM275401		Firmicutes	94	DQ057480	Bacterium ic1391	91,4	31	19	2	1	0	0	2	0	0	1	6
AM275403		Firmicutes	100	AJ270473	Butyrate-producing bacterium	100	8	4	1	1	1	0	0	0	0	0	1
AM275404		Firmicutes	100	AY442828	Bacterium YE64	95,4	2	1	0	1	0	0	0	0	0	0	0
AM275405		Firmicutes	94	AF445202	Swine fecal bacterium FPC67	94,6	22	14	4	2	0	0	0	0	0	2	0
AM275406		Firmicutes	46	AJ318889	Ruminococcus sp. 16442	84,9	9	9	0	0	0	0	0	0	0	0	0
AM275408		Firmicutes	100	AF519171	Lactobacillus gasseri strain ATCC 33323	100	16	15	0	0	0	1	0	0	0	0	0
AM275409		Firmicutes	100	Y18176	Clostridium disporicum	96,1	8	7	0	0	0	0	0	0	0	0	1
AM275410		Firmicutes	100	L34421	Eubacterium ventriosum	99,6	14	7	4	0	3	0	0	0	0	0	0
AM275411		Firmicutes	80	DQ057480	Bacterium ic1391	99,6	2	2	0	0	0	0	0	0	0	0	0
AM275412		Firmicutes	51	AB055907	Erysipelothrix rhusiopathiae	83,0	1	1	0	0	0	0	0	0	0	0	0
AM275413		Firmicutes	100	AB064904	Ruminococcus sp. CO47	99,4	101	12	55	12	6	1	0	0	3	2	10
AM275414		Firmicutes	49	AY733085	Lactobacillus oligofermentans strain 533	76,0	3	3	0	0	0	0	0	0	0	0	0
AM275415		Firmicutes	100	X73441	Clostridium spiroforme	98,9	2	1	0	0	0	0	0	0	0	1	0
AM275418		Firmicutes	68	AB075676	Firmicutes str. C34	83,6	6	6	0	0	0	0	0	0	0	0	0
AM275420		Firmicutes	100	AY305319	Butyrate-producing bacterium	99,6	30	13	10	1	2	1	0	0	0	3	0
AM275422		Firmicutes	100	L14675	Lachnospira pectinoschiza	93,8	3	3	0	0	0	0	0	0	0	0	0
AM275423		Firmicutes	88	Y18181	Clostridium methylpentosum	90,9	3	2	0	0	1	0	0	0	0	0	0
AM275429		Firmicutes	92	AB064882	Human intestinal firmicute CO31	89,2	2	2	0	0	0	0	0	0	0	0	0
AM275432		Firmicutes	100	L34621	Eubacterium halii	98,9	8	1	5	0	1	1	0	0	0	0	0
AM275434		Firmicutes	99	Y18188	Clostridium cocleatum	89,5	7	6	0	0	0	0	0	0	0	1	0
AM275435		Firmicutes	100	AY169415	Clostridium nexile	99,6	14	1	7	5	0	0	0	0	0	0	1
AM275437		Firmicutes	100	AJ417499	Lactobacillus farciminis	99,2	15	15	0	0	0	0	0	0	0	0	0
AM275438		Firmicutes	100	AY458860	Clostridium lituseburense	98,3	3	3	0	0	0	0	0	0	0	0	0
AM275440		Firmicutes	100	DQ445858	Clostridium sp. C01-2409	97,2	7	7	0	0	0	0	0	0	0	0	0
AM275441		Firmicutes	100	L14676	Roseburia cecicola	96,8	3	2	0	0	1	0	0	0	0	0	0
AM275445		Firmicutes	51	AJ229193	Unidentified eubacterium	83,3	6	6	0	0	0	0	0	0	0	0	0
AM275446		Firmicutes	100	AY438672	Clostridium bartlettii strain WAL 16138	99,5	4	4	0	0	0	0	0	0	0	0	0
AM275449		Firmicutes	62	AB064904	Ruminococcus sp. CO47	84,3	2	2	0	0	0	0	0	0	0	0	0
AM275452		Firmicutes	100	Y18176	Clostridium disporicum	97,7	4	4	0	0	0	0	0	0	0	0	0
AM275463		Firmicutes	75	Y18181	Clostridium methylpentosum	83,7	1	1	0	0	0	0	0	0	0	0	0
AM275466		Firmicutes	88	AB075681	Firmicutes str. C30	91,6	4	4	0	0	0	0	0	0	0	0	0
AM275467		Firmicutes	88	AB075681	Firmicutes str. C30	96,2	6	6	0	0	0	0	0	0	0	0	0
AM275472		Firmicutes	91	L34628	Eubacterium xylanophilum	93,0	2	1	0	1	0	0	0	0	0	0	0
AM275473		Firmicutes	50	AB064904	Ruminococcus sp. CO47	83,1	3	3	0	0	0	0	0	0	0	0	0
AM275474		Firmicutes	100	AB196512	Ruminococcus productus	94,3	3	3	0	0	0	0	0	0	0	0	0
AM275479		Firmicutes	100	AJ249539	Pediococcus acidilactici	99,8	2	2	0	0	0	0	0	0	0	0	0
AM275480		Firmicutes	91	AY699283	Lachnospira pectinoschiza isolate M56	89,5	1	1	0	0	0	0	0	0	0	0	0
AM275489		Firmicutes	100	AY169411	Ruminococcus obeum clone 1-4	97,6	14	1	5	5	2	0	0	0	0	0	1
AM275494		Firmicutes	100	AB064896	Ruminococcus sp. CO12	97,7	21	1	1	6	7	3	0	0	0	1	2
AM275497		Firmicutes	94	X89981	B.crossotus	98,0	17	9	5	1	1	0	0	0	0	0	1
AM275501		Firmicutes	98	AJ270471	Butyrate-producing bacterium	94,2	1	1	0	0	0	0	0	0	0	0	0
AM275505		Firmicutes	100	M59230	Eubacterium biforme	97,6	12	5	4	0	1	1	0	0	0	0	1
AM275507		Firmicutes	59	AJ229193	Unidentified eubacterium	81,5	2	2	0	0	0	0	0	0	0	0	0
AM275515		Firmicutes	70	AY949859	Clostridium sp. strain Z6	86,4	1	1	0	0	0	0	0	0	0	0	0
AM275519		Firmicutes	100	AY169422	Clostridium clostridioforme	99,4	38	2	4	7	10	7	0	1	4	2	1
AM275522		Firmicutes	100	AY960564	Ruminococcus sp. ID8	98,7	1	1	0	0	0	0	0	0	0	0	0

AM275539		Firmicutes	66	AY044915	Bulleidia moorei strain AHP 13983	88,6	1	1	0	0	0	0	0	0	0	0	0
AM275546		Firmicutes	99	AB075681	Firmicutes str. C30	90,5	1	1	0	0	0	0	0	0	0	0	0
AM275555		Firmicutes	100	L34619	Eubacterium formicigenerans	97,1	14	2	7	3	0	1	0	0	0	0	1
AM275560		Firmicutes	100	AY804150	Roseburia faecalis strain M88/1	99,4	49	3	3	11	16	5	3	0	2	3	3
AM275564		Firmicutes	100	AY305318	Butyrate-producing bacterium	98,9	4	1	1	0	0	1	0	0	0	0	1
AM275565		Firmicutes	98	AB080892	Clostridium sp. CJ6	94,2	1	1	0	0	0	0	0	0	0	0	0
AM275566		Firmicutes	100	AJ270475	Butyrate-producing bacterium	99,3	160	3	38	36	33	17	1	0	3	10	19
AM275568		Firmicutes	100	AY485605	Streptococcus parasanguinis	99,4	1	1	0	0	0	0	0	0	0	0	0
AM275571		Firmicutes	100	CP000023	Streptococcus thermophilus LMG 18311	100	12	2	4	0	1	1	0	0	0	3	1
AM275576		Firmicutes	100	AM157434	Enterococcus faecium	99,8	4	2	2	0	0	0	0	0	0	0	0
AM275577		Firmicutes	100	AY162469	Dialister invisus	99,8	18	1	2	6	3	1	0	0	0	2	3
AM275581		Firmicutes	100	AJ270479	Butyrate-producing bacterium	100	17	3	2	4	2	4	0	0	0	2	0
AM275582		Firmicutes	98	AY044915	Bulleidia moorei strain AHP 13983	86,2	1	1	0	0	0	0	0	0	0	0	0
AM275585		Firmicutes	99	AB064899	Ruminococcus sp. CB3	99,8	36	2	11	7	4	2	1	0	2	3	4
AM275587		Firmicutes	49	AB239468	Lactobacillus casei	76,4	3	3	0	0	0	0	0	0	0	0	0
AM275589		Firmicutes	95	L34418	Clostridium herbivorans	88,7	1	1	0	0	0	0	0	0	0	0	0
AM275600		Firmicutes	90	AF104839	Ruminococcus albus strain AR67	90,8	20	1	4	3	1	0	0	0	3	1	7
AM275604		Firmicutes	76	AY574991	Catabacter hongkongensis	86,0	1	1	0	0	0	0	0	0	0	0	0
AM275617		Firmicutes	54	AF262239	Clostridium leptum	77,8	2	1	1	0	0	0	0	0	0	0	0
AM275625		Firmicutes	99	AB064895	Ruminococcus sp. CO7	98,0	14	2	0	4	5	1	0	0	0	1	1
AM275657		Firmicutes	99	AY169414	Lachnospira pectinoschiza	100	1	1	0	0	0	0	0	0	0	0	0
AM275662		Firmicutes	100	AJ404682	Unidentified bacterium ZF5	95,8	2	1	1	0	0	0	0	0	0	0	0
AM275665		Firmicutes	99	AY305305	Butyrate-producing bacterium	90,2	1	1	0	0	0	0	0	0	0	0	0
AM275672		Firmicutes	100	AY323523	Peptostreptococcus micros	97,1	1	1	0	0	0	0	0	0	0	0	0
AM275673		Firmicutes	74	AF262239	Clostridium leptum	81,1	1	1	0	0	0	0	0	0	0	0	0
AM275681		Firmicutes	100	AJ270485	Butyrate-producing bacterium	99,6	16	1	2	8	2	0	0	0	1	1	1
AM275684		Firmicutes	92	M59230	Eubacterium biforme	90,2	1	1	0	0	0	0	0	0	0	0	0
AM275698		Firmicutes	100	AJ270491	Butyrate-producing bacterium	85,5	11	1	3	2	3	0	0	0	0	0	2
AM275701		Firmicutes	100	AY305319	Butyrate-producing bacterium	97,4	3	1	0	0	0	0	0	0	0	1	1
AM275714		Firmicutes	92	AY949859	Clostridium sp. strain Z6	86,5	1	1	0	0	0	0	0	0	0	0	0
AM275717		Firmicutes	95	AJ270471	Butyrate-producing bacterium	99,6	6	0	0	0	2	0	4	0	0	0	0
AM275719		Firmicutes	100	AY169429	Faecalibacterium prausnitzii	96,9	6	0	0	0	1	0	1	2	0	2	0
AM275720		Firmicutes	97	AJ270475	Butyrate-producing bacterium	91,9	1	0	0	0	1	0	0	0	0	0	0
AM275721		Firmicutes	98	AF357567	Bacterium mpn-isolate group 19	96,6	2	0	0	0	1	0	0	0	0	0	1
AM275726		Firmicutes	100	AJ270469	Butyrate-producing bacterium	96,9	6	0	0	0	1	0	2	0	0	3	0
AM275729		Firmicutes	80	AY442821	Bacterium YE57	85,2	2	0	0	0	1	0	1	0	0	0	0
AM275731		Firmicutes	93	DQ168656	Clostridiales bacterium JN18_V41_S	85,9	1	0	0	0	1	0	0	0	0	0	0
AM275732		Firmicutes	100	AY169429	Faecalibacterium prausnitzii	96,7	7	0	0	0	1	0	4	0	0	1	1
AM275733		Firmicutes	100	DQ057458	Bacterium ic1294	99,8	1	0	0	0	1	0	0	0	0	0	0
AM275734		Firmicutes	86	L34618	Eubacterium desmolans	86,3	2	0	0	0	1	0	0	0	0	0	1
AM275735		Firmicutes	100	DQ479412	Clostridium sp. CYP2	95,9	2	0	0	0	1	0	0	0	0	0	1
AM275739		Firmicutes	90	AY442821	Bacterium YE57	85,4	11	0	0	0	1	5	2	0	0	2	1
AM275741		Firmicutes	83	AY442821	Bacterium YE57	85,6	1	0	0	0	1	0	0	0	0	0	0
AM275754		Firmicutes	100	AY169429	Faecalibacterium prausnitzii	96,3	5	0	0	0	1	1	1	0	0	2	0
AM275756		Firmicutes	100	AY277937	Streptococcus constellatus strain 1	100	2	0	0	0	1	0	0	0	0	1	0
AM275757		Firmicutes	96	DQ278862	Clostridium aminophilum isolate 152R-1b	90,8	1	0	0	0	1	0	0	0	0	0	0
AM275758		Firmicutes	100	AB080895	Human intestinal firmicute CJ7	99,6	3	0	0	0	1	1	0	1	0	0	0
AM275760		Firmicutes	100	AB064892	Ruminococcus sp. CO41	99,8	1	0	0	0	1	0	0	0	0	0	0
AM275763		Firmicutes	84	AY136666	Bacteroides capillosus	89,1	1	0	0	0	1	0	0	0	0	0	0
AM275764		Firmicutes	83	AY487928	Acetanaerobacterium elongatum strain Z7	82,3	1	0	0	0	1	0	0	0	0	0	0
AM275771		Firmicutes	93	AF262236	Eubacterium sp. VPI 12708	100	1	0	0	0	1	0	0	0	0	0	0
AM275784		Firmicutes	100	DQ057460	Bacterium ic1297	96,1	1	0	0	0	1	0	0	0	0	0	0
AM275785		Firmicutes	97	AY169411	Ruminococcus obeum clone 1-4	97,6	2	0	0	0	2	0	0	0	0	0	0
AM275787		Firmicutes	99	X71855	C.xylanolyticum	92,7	1	0	0	0	1	0	0	0	0	0	0
AM275790		Firmicutes	100	AJ270469	Butyrate-producing bacterium	98,5	42	0	0	0	3	12	10	1	3	5	8
AM275792		Firmicutes	100	AY305307	Butyrate-producing bacterium	99,6	21	0	0	0	1	12	3	1	0	4	0
AM275794		Firmicutes	100	AB064906	Human intestinal firmicute CO19	98,9	26	0	0	0	1	7	11	0	0	6	1
AM275797		Firmicutes	100	AJ305238	Clostridium leptum	100	4	0	0	0	2	2	0	0	0	0	0
AM275799		Firmicutes	100	AJ508452	Clostridium boltei	95,5	4	0	0	0	3	0	0	0	0	0	1
AM275806		Firmicutes	100	AJ270469	Butyrate-producing bacterium	99,6	42	0	0	0	4	4	15	1	2	10	6
AM275807		Firmicutes	100	AJ270474	Butyrate-producing bacterium	95,1	2	0	0	0	2	0	0	0	0	0	0
AM275812		Firmicutes	100	AY305309	Butyrate-producing bacterium	95,1	4	0	0	0	2	2	0	0	0	0	0
AM275814		Firmicutes	100	X85100	R.callidus	99,8	2	0	0	0	2	0	0	0	0	0	0
AM275816		Firmicutes	100	X76739	Clostridium xylanolyticum	95,8	7	0	0	0	4	2	0	0	0	0	1
AM275817		Firmicutes	100	AB064892	Ruminococcus sp. CO41	95,7	1	0	0	0	1	0	0	0	0	0	0
AM275820		Firmicutes	99	AB125231	Ruminococcus sp. M-1	94,5	8	0	0	0	5	2	0	0	1	0	0
AM275822		Firmicutes	100	AJ508452	Clostridium boltei	94,4	8	0	0	0	4	2	0	0	1	1	0
AM275828		Firmicutes	78	AB239487	Rumen bacterium R-21	83,5	13	0	0	0	5	2	0	0	0	1	5
AM275830		Firmicutes	99	AF548373	Allisonella histaminiformans	88,2	5	0	0	0	2	0	0	0	0	2	1
AM275858		Firmicutes	100	AB064909	Fusobacterium sp. CO6	99,1	9	0	0	0	1	2	4	1	0	1	0
AM275866		Firmicutes	100	AJ270470	Butyrate-producing bacterium	98,8	9	0	0	0	1	2	2	0	0	3	1
AM275881		Firmicutes	100	AY350746	Butyrate-producing bacterium	99,3	2	0	0	0	2	0	0	0	0	0	0
AM275968		Firmicutes	100	AB064892	Ruminococcus sp. CO41	95,8	2	0	0	0	2	0	0	0	0	0	0
AM276030		Firmicutes	95	AJ270471	Butyrate-producing bacterium	95,8	1	0	0	0	1	0	0	0	0	0	0
AM276067		Firmicutes	87	AY136666	Bacteroides capillosus	93,5	2	0	0	0	0	0	0	1	0	1	0
AM276069		Firmicutes	84	AY442821	Bacterium YE57	85,9	2	0	0	0	0	0	0	1	0	1	0
AM276070		Firmicutes	84	AB040498	Oscillospira guilliermondii	87,1	1	0	0	0	0	0	0	1	0	0	0
AM276084		Firmicutes	78	Z49863	T.aceticus	87,2	1	0	0	0	0	0	0	1	0	0	0
AM276089		Firmicutes	95	AB239481	Rumen bacterium R-7	87,1	1	0	0	0	0	0	0	1	0	0	0
AM276094		Firmicutes	82	AB080898	Human intestinal firmicute CJ6	94,3	1	0	0	0	0	0	0	1	0	0	0
AM404446		Firmicutes	100	Y18176	Clostridium disporicum	98,4	2	0	1	0	1	0	0	0	0	0	0
AM404447		Firmicutes	55	AJ270474	Butyrate-producing bacterium	94,2	1	0	1	0	0	0	0	0	0	0	0
AM404448		Firmicutes	100	AJ270474	Butyrate-producing bacterium	94,2	3	0	1	1	0	0	0	0	0	1	0
AM404449		Firmicutes	100	AJ508452	Clostridium boltei, strain 16351	95,7	1	0	1	0	0	0	0	0	0	0	0
AM404451		Firmicutes	100	AJ270475	Butyrate-producing bacterium A1-86	97,1	1	0	1	0	0	0	0	0	0	0	0
AM404452		Firmicutes	100	AY995769	Veillonella parvula strain ATCC 17745	99,8	1	0	1	0	0	0	0	0	0	0	0
AM404453		Firmicutes	100	Y18185	Clostridium saccharolyticum	93,5	2	0	2	0	0	0	0	0	0	0	0
AM404454		Firmicutes	100	AJ270484	Butyrate-producing bacterium	97,5	1	0	1	0	0	0	0	0	0	0	0
AM404455		Firmicutes	100	L34623	Eubacterium ramulus	98,9	10	0	1	5	2	0	0	0	0	0	2
AM404457		Firmicutes	78	AF445202	Swine fecal bacterium FPC67	91,1	3	0	1	0	1	0	0	0	0	0	1
AM404458		Firmicutes	100	AJ270491	Butyrate-producing bacterium	99,5	7	0	2	2	1	1	0	0	1	0	0
AM404459		Firmicutes	99	X72865	P.faecium (ACM3679)	88,0	3	0	1	1	1	0	0	0	0	0	0
AM404460		Firmicutes	91	AF030449	Ruminococcus flavefaciens strain ATCC 49949	93,8	1	0	1	0	0	0	0	0	0	0	0
AM404461	X	Firmicutes	93	AB064938	Clostridium sp. CS9	91,5	1	0	1	0	0	0	0	0	0	0	0
AM404465	X	Firmicutes	69	AB239485	Rumen bacterium R-15	87,3	4	0	2	2	0	0	0	0	0	0	0
AM404466		Firmicutes	100	AY305306	Butyrate-producing bacterium	99,8	3	0	2	0	1	0	0	0	0	0	0
AM404467		Firmicutes	100	AF445200	Swine fecal bacterium FPC73	99,3	27	0	3	9	6	2	0	0	2	3	2
AM404468		Firmicutes	99	AY169411	Ruminococcus obeum clone 1-4	94,4	3	0	1	2	0	0	0	0	0	0	0
AM404469		Firmicutes	98	AJ318890	Clostridium sp. 14505	93,5	1	0	1	0	0	0	0	0	0	0	0
AM404470		Firmicutes	99	L34418	Clostridium herbivorans	92,3	4	0	1	0	1	1	0	0	0	1	0
AM404473		Firmicutes	100	AJ518873	Lachnobacterium sp. wal 14165	96,2	2	0	2	0	0	0	0	0	0	0	0
AM404474		Firmicutes	100	AJ938182	Staphylococcus aureus RF122	99,8	1	0	1	0	0	0	0	0	0	0	0
AM404475		Firmicutes	100	AY169429	Faecalibacterium prausnitzii	95,4	1	0	1	0	0	0	0	0	0	0	0
AM404477	X	Firmicutes	100	DQ057460	Bacterium ic1297	94,4	1	0	1	0	0	0	0	0	0	0	0
AM404478		Firmicutes	100	AY960572	Clostridium sp. ID6	93,7	16	0	2	7	4	2	0	0	0	0	1
AM404480		Firmicutes	100	AB064890	Human intestinal firmicute CO19	98,0	42	0	17	11	6	2	0	0	0	3	3
AM404481		Firmicutes	100	AY305305	Butyrate-producing bacterium	99,3	14	0	2	2	3	1	1	0	0	2	3
AM404482		Firmicutes	100	AB064891	Ruminococcus sp. CO28	100	1	0	1	0	0	0	0	0	0	0	0
AM404486		Firmicutes	94	AF202259	Eubacterium oxidoreducens strain G2-2	90,6	1	0	1	0	0	0	0	0	0	0	0
AM404487		Firmicutes	94	AF445207	Swine fecal bacterium FPC110	88,4	1	0	1	0	0	0	0	0	0	0	0
AM404494		Firmicutes	100	M59114	Streptococcus hansenii	97,5	2	0	2	0	0	0	0	0	0	0	0
AM404497		Firmicutes	97	AY169414	Lachnospira pectinoschiza	96,4	4	0	3	0	1	0	0	0	0	0	0
AM404499		Firmicutes	96	AB064900	Ruminococcus sp. CO27	98,6	10	0	2	3	2	0	1	0	0	1	1
AM404500		Firmicutes	100	AB080894	Ruminococcus sp. CJ63	98,7	6	0	2	3	1	0	0	0	0	0	0
AM404501	X	Firmicutes	89	AB239485	Rumen bacterium R-15	88,1	4	0	1	1	0	1	0	0	0	1	0
AM404502		Firmicutes	99	AB064900	Ruminococcus sp. CO27	99,1	22	0	2	11	5	1	1	0	0	1	1
AM404506		Firmicutes	100	AB075679	Ruminococcus sp. C36	96,8	1	0	1	0	0	0	0	0	0	0	0
AM404510		Firmicutes	100	AF357567	Bacterium mpn-isolate group group 19	98,2	6	0	2	4	0	0	0	0	0	0	0
AM404511		Firmicutes	99	AB222208	Ruminococcus sp. K-1	99,8	46	0	12	16	5	3	0	0	3	2	5
AM404512		Firmicutes	100	L34420	Eubacterium eligens	99,1	10	0	6	1	0	0	0	0	0	2	1
AM404515		Firmicutes	99	AB064895	Ruminococcus sp. CO7	97,3	3	0	3	0	0	0	0	0	0	0	0
AM404516		Firmicutes	96	AB064891	Ruminococcus sp. CO28	90,6	1	0	1	0	0	0	0	0	0	0	0
AM404518		Firmicutes	100	Y10584	Clostridium sp.	93,8	1	0	1	0	0	0	0	0	0	0	0
AM404519		Firmicutes	100	L34619	Eubacterium formicigenerans	94,4	3	0	3	0	0	0	0	0	0	0	0
AM404523		Firmicutes	92	AF116920	Clostridium xylanovorans	90,6	1	0	1	0	0	0	0	0	0	0	0
AM404524		Firmicutes	76	AB040498	Oscillospira guilliermondii	84,9	2	0	1	0	0	0	0	0	0	1	0
AM404525		Firmicutes	100	U13039	Eubacterium infirmum W 1471	88,0	7	0	1	1	3	0	0	0	0	2	0
AM404526		Firmicutes	100	AY169414	Lachnospira pectinoschiza	97,5	2	0	1	1	0	0	0	0	0	0	0
AM404532		Firmicutes	100	AF357572	Bacterium mpn-isolate group 24	98,0	12	0	5	4	2	0	0	0	0	0	1
AM404534		Firmicutes	100	L76604	Ruminococcus torques	100	11	0	5	4	1	0	0	0	0	0	1
AM404537		Firmicutes	83	L34625	Eubacterium siraeum	89,4	4	0	1	0	1	1	0	0	1	0	0
AM404539		Firmicutes	92	AF030449	Ruminococcus flavefaciens strain ATCC 49949	93,5	2	0	1	1	0	0	0	0	0	0	0
AM404540		Firmicutes	100	AY305305	Butyrate-producing bacterium	96,5	2	0	1	1	0	0	0	0	0	0	0
AM404541		Firmicutes	100	AB008552	Eubacterium ruminantium	90,5	1	0	1	0	0	0	0	0	0	0	0
AM404542		Firmicutes	100	AB064901	Ruminococcus sp. CB10	99,1	14	0	5	4	2	0	0	0	0	1	2
AM404543		Firmicutes	100	AB064891	Ruminococcus sp. CO28	95,9	2	0	2	0	0	0	0	0	0	0	0
AM404544		Firmicutes	100	L76597	Ruminococcus gnavus	99,8	2	0	1	1	0	0	0	0	0	0	0
AM404546		Firmicutes	100	AF357572	Bacterium mpn-isolate group 24	99,8	20	0	8	5	4	0	0	0	0	1	2
AM404548		Firmicutes	98	AJ270491	Butyrate-producing bacterium	90,5	3	0	1	1	1	0	0	0	0	0	0
AM404549		Firmicutes	99	AF070465	Mixed culture ruminal bacterium PAD160	85,6	1	0	1	0	0	0	0	0	0	0	0
AM404551		Firmicutes	100	AB064890	Human intestinal firmicute CB17	99,8	18	0	5	5	3	0	0	0	0	1	4
AM404607		Firmicutes	100	AY169411	Ruminococcus obeum clone 1-4	98,7	17	0	3	4	4	4	0	0	0	2	0
AM404797		Firmicutes	97	AB064900	Ruminococcus sp. CO27	98,0	6	0	0	1	1	0	0	0	0	1	3
AM404798		Firmicutes	100	AJ270475	Butyrate-producing bacterium	96,4	1	0	0	1	0	0	0	0	0	0	0
AM404808		Firmicutes	100	AY033434	Clostridium lactatifermentan	99,3	2	0	0	1	1	0	0	0	0	0	0
AM404810		Firmicutes	100	AB064934	Human intestinal firmicute CB12	98,8	3	0	0	1	1	1	0	0	0	0	0
AM404814		Firmicutes	88	AB040498	Oscillospira guilliermondii	87,2	5	0	0	1	0	1	1	1	0	0	1
AM404819		Firmicutes	100	AY305322	Butyrate-producing bacterium	93,1	2	0	0	1	0	0	0	0	0	1	0
AM404820		Firmicutes	100	AJ270469	Butyrate-producing bacterium	97,2	13	0	0	1	2	4	2	0	1	1	2
AM404826		Firmicutes	100	AJ270485	Butyrate-producing bacterium	97,5	1	0	0	1	0	0	0	0	0	0	0
AM404830	X	Firmicutes	100	X76739	Clostridium xylanolyticum	92,2	1	0	0	1	0	0	0	0	0	0	0
AM404831		Firmicutes	94	AB080893	Clostridium sp. CJ23	91,1	1	0	0	1	0	0	0	0	0	0	0
AM404838		Firmicutes	87	Y18181	Clostridium methylpentosum	86,5	3	0	0	2	0	0	0	0	0	1	0
AM404840		Firmicutes	94	Z49863	T.aceticus	85,0	6	0	0	1	0	0	3	0	0	2	0
AM404844		Firmicutes	99	AB064900	Ruminococcus sp. CO27	96,9	3	0	0	1	1	0	0	0	0	1	0
AM404845		Firmicutes	93	AJ270471	Butyrate-producing bacterium	95,4	3	0	0	1	1	0	1	0	0	0	0
AM404846		Firmicutes	100	X94964	Ruminococcus schinkii	93,8	1	0	0	1	0	0	0	0	0	0	0
AM404847	X	Firmicutes	91	AB196512	Ruminococcus productus	86,5	1	0	0	1	0	0	0	0	0	0	0
AM404849		Firmicutes	99	L34625	Eubacterium siraeum	99,3	6	0	0	2	3	0	0	1	0	0	0
AM404852		Firmicutes	100	AJ508452	Clostridium boltei	94,2	1	0	0	1	0	0	0	0	0	0	0

AM404855	X	Firmicutes	98	AJ518873	Lachnobacterium sp. wal 1416	94,0	1	0	0	1	0	0	0	0	0	0	0	0
AM404865		Firmicutes	100	AJ508452	Clostridium boltei	94,7	1	0	0	1	0	0	0	0	0	0	0	0
AM404871		Firmicutes	100	AY937379	Ruminococcus productus strain SECO-Mt75m3	93,7	3	0	0	1	2	0	0	0	0	0	0	0
AM404876		Firmicutes	100	AY350746	Butyrate-producing bacterium	98,9	35	0	0	6	20	4	0	0	0	0	2	3
AM404890		Firmicutes	100	AY169427	Faecalibacterium prausnitzii	99,5	116	0	0	2	15	37	18	1	3	20	20	
AM404892		Firmicutes	100	AY350746	Butyrate-producing bacterium	91,6	3	0	0	1	0	1	0	0	0	1	0	
AM404895		Firmicutes	100	AB075678	Firmicutes str. C38	95,7	4	0	0	1	0	0	0	0	1	0	2	
AM404896		Firmicutes	100	AB008552	Eubacterium ruminantium	89,8	1	0	0	1	0	0	0	0	0	0	0	
AM404899		Firmicutes	93	DQ168656	Clostridiales bacterium JN18_V41_S	87,7	1	0	0	1	0	0	0	0	0	0	0	
AM404900		Firmicutes	99	AB080900	Clostridium sp. CJ66	99,3	1	0	0	1	0	0	0	0	0	0	0	
AM404901		Firmicutes	97	AY169422	Clostridium clostridioforme	97,5	8	0	0	1	2	1	0	0	1	1	2	
AM404902		Firmicutes	98	AB064895	Ruminococcus sp. CO7	99,1	14	0	0	4	4	0	0	0	0	3	3	
AM404903		Firmicutes	99	AY305322	Butyrate-producing bacterium	93,6	4	0	0	1	2	0	0	0	0	0	1	
AM404912		Firmicutes	98	AB064900	Ruminococcus sp. CO27	97,3	2	0	0	1	0	0	0	0	1	0	0	
AM404918		Firmicutes	100	AY169411	Ruminococcus obeum clone 1-4	97,3	20	0	0	2	10	2	0	0	0	2	4	
AM405040		Firmicutes	98	AB064895	Ruminococcus sp. CO7	99,8	5	0	0	3	0	0	0	0	0	2	0	
AM405109		Firmicutes	72	AB080896	Human intestinal firmicute CJ36	87,6	1	0	0	0	0	1	0	0	0	0	0	
AM405111		Firmicutes	94	Z49863	T.aceticus	87,5	2	0	0	0	0	1	0	0	0	1	0	
AM405112	X	Firmicutes	77	AB080895	Human intestinal firmicute CJ7	84,1	1	0	0	0	0	1	0	0	0	0	0	
AM405114		Firmicutes	95	AF150697	Elbe River snow isolate Iso15	91,1	1	0	0	0	0	1	0	0	0	0	0	
AM405115		Firmicutes	100	AJ305238	Clostridium leptum	97,8	1	0	0	0	0	1	0	0	0	0	0	
AM405116		Firmicutes	81	Z49863	T.aceticus	86,1	1	0	0	0	0	1	0	0	0	0	0	
AM405117	X	Firmicutes	91	AJ310082	Clostridium stercorarium	84,1	1	0	0	0	0	1	0	0	0	0	0	
AM405119		Firmicutes	100	AJ270482	Butyrate-producing bacterium	99,8	1	0	0	0	0	1	0	0	0	0	0	
AM405120		Firmicutes	75	AF087643	Bacterium PS7	83,3	1	0	0	0	0	1	0	0	0	0	0	
AM405122		Firmicutes	82	AB080897	Human intestinal firmicute CJ31	89,4	3	0	0	0	0	1	0	0	0	2	0	
AM405126	X	Firmicutes	94	DQ057456	Bacterium ic1291	91,3	1	0	0	0	0	1	0	0	0	0	0	
AM405127	X	Firmicutes	56	L34682	Eubacterium dolichum	86,6	1	0	0	0	0	1	0	0	0	0	0	
AM405131		Firmicutes	87	AF157051	Bacterium ASF500	88,0	5	0	0	0	0	1	0	0	0	3	1	
AM405135	X	Firmicutes	86	AY442821	Bacterium YE57	87,8	4	0	0	0	0	1	1	0	0	0	2	
AM405136		Firmicutes	100	AY169429	Faecalibacterium prausnitzii	97,0	5	0	0	0	0	1	2	1	0	1	0	
AM405139		Firmicutes	73	AF097316	Unidentified bacterium Par64	88,1	2	0	0	0	0	1	1	0	0	0	0	
AM405143		Firmicutes	93	Z49863	T.aceticus	86,3	1	0	0	0	0	1	0	0	0	0	0	
AM405145		Firmicutes	89	AY949857	Clostridium sp. strain P6	85,8	1	0	0	0	0	1	0	0	0	0	0	
AM405148		Firmicutes	100	U95028	Megasphaera elsdenii S2	99,2	2	0	0	0	0	2	0	0	0	0	0	
AM405156	X	Firmicutes	89	AB125279	Clostridium straminisolvens	84,7	1	0	0	0	0	1	0	0	0	0	0	
AM405158		Firmicutes	99	AY169429	Faecalibacterium prausnitzii	97,2	2	0	0	0	0	2	0	0	0	0	0	
AM405167	X	Firmicutes	55	AB239485	Rumen bacterium R-15	84,5	1	0	0	0	0	1	0	0	0	0	0	
AM405170	X	Firmicutes	85	AY487928	Acetanaerobacterium elongatum strain Z7	83,0	1	0	0	0	0	1	0	0	0	0	0	
AM405176		Firmicutes	100	AB064906	Human intestinal firmicute CO19	98,1	46	0	0	0	0	8	14	8	1	11	4	
AM405179	X	Firmicutes	97	AB080895	Human intestinal firmicute CJ7	92,7	1	0	0	0	0	1	0	0	0	0	0	
AM405183		Firmicutes	100	AJ270473	Butyrate-producing bacterium	93,8	1	0	0	0	0	1	0	0	0	0	0	
AM405188		Firmicutes	71	AY442821	Bacterium YE57	86,2	9	0	0	0	0	2	3	1	0	1	2	
AM405190		Firmicutes	90	AY581272	Lactobacillales bacterium HY-36-1	88,2	2	0	0	0	0	1	0	0	0	0	1	
AM405192		Firmicutes	100	Y11466	H.filiformis	99,7	1	0	0	0	0	1	0	0	0	0	0	
AM405199		Firmicutes	100	AY162469	Dialister invisus	89,9	1	0	0	0	0	1	0	0	0	0	0	
AM405201		Firmicutes	95	AJ270471	Butyrate-producing bacterium	97,6	4	0	0	0	0	2	2	0	0	0	0	
AM405207		Firmicutes	88	AB040498	Oscillospira guilliermondii	91,8	1	0	0	0	0	1	0	0	0	0	0	
AM405209		Firmicutes	88	AY949859	Clostridium sp. strain Z6	91,1	1	0	0	0	0	1	0	0	0	0	0	
AM405213		Firmicutes	100	AY169427	Faecalibacterium prausnitzii	96,2	3	0	0	0	0	2	0	0	0	1	0	
AM405215		Firmicutes	100	AJ508452	Clostridium boltei, strain 16351	94,2	1	0	0	0	0	1	0	0	0	0	0	
AM405217		Firmicutes	100	AJ270470	Butyrate-producing bacterium	98,8	42	0	0	0	0	6	18	2	2	7	7	
AM405221		Firmicutes	87	Y18187	Clostridium orbiscindens	89,2	2	0	0	0	0	1	0	0	0	1	0	
AM405223		Firmicutes	66	AY442821	Bacterium YE57	86,5	7	0	0	0	0	2	4	1	0	0	0	
AM405226		Firmicutes	78	AY442821	Bacterium YE57	85,5	2	0	0	0	0	2	0	0	0	0	0	
AM405229		Firmicutes	99	AY169429	Faecalibacterium prausnitzii	96,0	6	0	0	0	0	2	3	1	0	0	0	
AM405232		Firmicutes	100	AY305307	Butyrate-producing bacterium	98,4	8	0	0	0	0	2	1	1	0	2	2	
AM405233		Firmicutes	62	AJ229190	Unidentified eubacterium	85,9	3	0	0	0	0	3	0	0	0	0	0	
AM405258		Firmicutes	80	AY442821	Bacterium YE57	86,0	3	0	0	0	0	2	1	0	0	0	0	
AM405267		Firmicutes	70	AY442821	Bacterium YE57	86,3	35	0	0	0	0	14	14	0	0	6	1	
AM405302		Firmicutes	72	AY442821	Bacterium YE57	85,4	8	0	0	0	0	3	1	0	1	0	3	
AM405308		Firmicutes	100	AY169429	Faecalibacterium prausnitzii	97,4	1	0	0	0	0	1	0	0	0	0	0	
AM405344		Firmicutes	83	DQ286651	Bacterium AN1045	92,7	7	0	0	0	0	2	3	0	0	2	0	
AM405425	X	Firmicutes	87	AB239481	Rumen bacterium R-7	89,8	1	0	0	0	0	0	1	0	0	0	0	
AM405428		Firmicutes	100	AJ270469	Butyrate-producing bacterium	98,6	1	0	0	0	0	0	1	0	0	0	0	
AM405430		Firmicutes	83	AY574991	Catabacter hongkongensis	85,4	1	0	0	0	0	0	1	0	0	0	0	
AM405431		Firmicutes	92	AY445616	Bacterium 10C29	96,4	1	0	0	0	0	0	1	0	0	0	0	
AM405441		Firmicutes	75	AB080895	Human intestinal firmicute CJ7	84,9	2	0	0	0	0	0	2	0	0	0	0	
AM405446	X (AM405620)	Firmicutes	64	AY422354	Anaerobic bacterium U3/B-2	86,3	2	0	0	0	0	0	2	0	0	0	0	
AM405457		Firmicutes	80	AY136666	Bacteroides capillosus	94,8	2	0	0	0	0	0	1	1	0	0	0	
AM405466		Firmicutes	100	AY169429	Faecalibacterium prausnitzii	97,6	2	0	0	0	0	0	2	0	0	0	0	
AM405469		Firmicutes	95	DQ002932	Anaerotruncus colihominis strain HKU19	87,7	2	0	0	0	0	0	2	0	0	0	0	
AM405501		Firmicutes	82	AY442821	Bacterium YE57	89,6	2	0	0	0	0	0	2	0	0	0	0	
AM405507	X	Firmicutes	73	AF445207	Swine fecal bacterium FPC110	86,8	1	0	0	0	0	0	1	0	0	0	0	
AM405691		Firmicutes	100	AB064910	Human intestinal firmicute	98,8	7	0	0	0	0	2	2	1	0	1	1	
AM405718		Firmicutes	68	AB080895	Human intestinal firmicute CJ7	82,8	1	0	0	0	0	0	0	0	1	0	0	
AM405721		Firmicutes	82	AB080898	Human intestinal firmicute CJ6	95,3	1	0	0	0	0	0	0	0	1	0	0	
AM405736		Firmicutes	79	AB040498	Oscillospira guilliermondii	89,3	1	0	0	0	0	0	0	0	1	0	0	
AM405741		Firmicutes	52	AJ229193	Unidentified eubacterium	82,3	2	0	0	0	0	0	0	0	1	0	1	
AM406022		Firmicutes	89	AB080895	Human intestinal firmicute CJ7	88,2	1	0	0	0	0	0	0	0	0	1	0	
AM406028		Firmicutes	100	AF061009	Enterococcus cecorum	99,1	1	0	0	0	0	0	0	0	0	1	0	
AM406044		Firmicutes	97	AF167711	Papillibacter cinnaminovorans	88,0	1	0	0	0	0	0	0	0	0	1	0	
AM406047	X	Firmicutes	100	AF481209	Dialister sp. E2_20 E1	90,3	1	0	0	0	0	0	0	0	0	1	0	
AM406049		Firmicutes	100	L14326	Gemella haemolysans	98,7	1	0	0	0	0	0	0	0	0	1	0	
AM406050		Firmicutes	100	AY699273	Butyrivibrio fibrisolvens	89,1	1	0	0	0	0	0	0	0	0	1	0	
AM406051		Firmicutes	100	DQ149245	Lactococcus lactis	100	1	0	0	0	0	0	0	0	0	1	0	
AM406054		Firmicutes	99	AB064903	Human intestinal firmicute CO2	98,0	1	0	0	0	0	0	0	0	0	1	0	
AM406061	X	Firmicutes	82	Z49863	T.aceticus	86,3	1	0	0	0	0	0	0	0	0	1	0	
AM406067		Firmicutes	92	AJ270471	Butyrate-producing bacterium	96,5	1	0	0	0	0	0	0	0	0	1	0	
AM406073		Firmicutes	99	AY305319	Butyrate-producing bacterium	94,4	1	0	0	0	0	0	0	0	0	1	0	
AM406087		Firmicutes	82	AY136666	Bacteroides capillosus	94,9	1	0	0	0	0	0	0	0	0	1	0	
AM406093		Firmicutes	100	L34628	Eubacterium xylanophilum	88,2	2	0	0	0	0	0	0	0	0	2	0	
AM406099		Firmicutes	100	AE005672	Streptococcus pneumoniae TIGR4	99,8	3	0	0	0	0	0	0	0	0	3	0	
AM406134		Firmicutes	84	Y18187	Clostridium orbiscindens	88,8	2	0	0	0	0	0	0	0	0	1	1	
AM406234		Firmicutes	100	AY169429	Faecalibacterium prausnitzii	96,4	3	0	0	0	0	0	0	0	0	1	2	
AM406384		Firmicutes	52	AY442821	Bacterium YE57	85,5	1	0	0	0	0	0	0	0	0	0	1	
AM406399		Firmicutes	100	AJ518869	Subdoligranulum variabile, type strain BI 114T	98,0	1	0	0	0	0	0	0	0	0	0	1	
AM406400		Firmicutes	100	X89976	B.fibrisolvens	91,1	1	0	0	0	0	0	0	0	0	0	1	
AM406412		Firmicutes	100	AY169427	Faecalibacterium prausnitzii	96,2	1	0	0	0	0	0	0	0	0	0	1	
AM406417		Firmicutes	100	AF445206	Swine fecal bacterium FPC63	99,3	1	0	0	0	0	0	0	0	0	0	1	
AM406425		Firmicutes	100	CP000023	Streptococcus thermophilus LMG 18311	98,9	1	0	0	0	0	0	0	0	0	0	1	
AM406433		Firmicutes	98	L76597	Ruminococcus gnavus	92,0	1	0	0	0	0	0	0	0	0	0	1	
AM406449		Firmicutes	100	AJ270469	Butyrate-producing bacterium	94,9	1	0	0	0	0	0	0	0	0	0	1	
AM406451		Firmicutes	100	AY005045	Streptococcus mitis bv2	99,1	1	0	0	0	0	0	0	0	0	0	1	
AM406458	X	Firmicutes	74	AB080897	Human intestinal firmicute CJ31	91,6	1	0	0	0	0	0	0	0	0	0	1	
AM406496	X	Firmicutes	72	AY445616	Bacterium 10C29	92,7	1	0	0	0	0	0	0	0	0	0	1	
AM406499	X	Firmicutes	81	AB080898	Human intestinal firmicute CJ6	87,3	2	0	0	0	0	0	0	0	0	0	2	
AM406655		Firmicutes	100	AJ270470	Butyrate-producing bacterium	98,5	1	0	0	0	0	0	0	0	0	0	1	
AM275436		Proteobacteria	37	AF404726	Pediococcus dextrinicus clone P17	78,0	5	5	0	0	0	0	0	0	0	0	0	
AM275469		Proteobacteria	51	AF349724	Gram-positive bacterium MOL361	81,9	2	2	0	0	0	0	0	0	0	0	0	
AM275475		Proteobacteria	57	AF404726	Pediococcus dextrinicus clone P17	77,7	7	7	0	0	0	0	0	0	0	0	0	
AM275637		Proteobacteria	100	AY362908	Haemophilus parainfluenzae strain CCUG 12836	98,5	2	1	1	0	0	0	0	0	0	0	0	
AM275742		Proteobacteria	100	U49756	Oxalobacter formigenes	99,1	1	0	0	0	1	0	0	0	0	0	0	
AM275746		Proteobacteria	100	AE014075	Escherichia coli CFT073	99,2	1	0	0	0	1	0	0	0	0	0	0	
AM404827		Proteobacteria	93	AL646052	Ralstonia solanacearum GMI1000	85,2	3	0	0	1	1	1	0	0	0	0	0	
AM405146	X	Proteobacteria	100	L37785	Sutterella wadsworthensis strain 7877	92,5	2	0	0	0	0	2	0	0	0	0	0	
AM405171		Proteobacteria	100	L37785	Sutterella wadsworthensis strain 7877	99,7	1	0	0	0	0	1	0	0	0	0	0	
AM405451		Proteobacteria	100	AF192152	Desulfomonas pigra	99,8	4	0	0	0	0	0	1	0	3	0	0	
AM405461		Proteobacteria	95	DQ168657	Beta proteobacterium JN18_A91	90,5	1	0	0	0	0	0	1	0	0	0	0	
AM405481		Proteobacteria	100	U82813	Bilophila wadsworthia	98,6	4	0	0	0	0	0	3	0	0	1	0	
AM405762		Proteobacteria	100	AE004844	Pseudomonas aeruginosa PAO1	99,7	2	0	0	0	0	0	0	0	2	0	0	
AM405776		Proteobacteria	100	AM040265	Brucella melitensis biovar Abortus	99,3	4	0	0	0	0	0	0	0	2	1	1	
AM406025		Proteobacteria	100	DQ329348	Bacterium C30	98,6	1	0	0	0	0	0	0	0	0	1	0	
AM406046	X	Proteobacteria	61	M94280	Polyangium sp.	84,0	1	0	0	0	0	0	0	0	0	1	0	
AM406068		Proteobacteria	100	AJ001345	thin bent rods	99,7	1	0	0	0	0	0	0	0	0	1	0	
AM406069		Proteobacteria	100	AJ888983	Acinetobacter calcoaceticus	100	1	0	0	0	0	0	0	0	0	1	0	
AM406106		Proteobacteria	100	AF149849	Delftia acidovorans strain MC1	99,5	5	0	0	0	0	0	0	0	0	2	3	
AM406383		Proteobacteria	79	BA000045	Gloeobacter violaceus PCC 7421	85,6	1	0	0	0	0	0	0	0	0	0	1	
AM406390		Proteobacteria	100	CP000243	Escherichia coli UTI89	99,8	1	0	0	0	0	0	0	0	0	0	1	
AM406391		Proteobacteria	100	AJ833647	Byssophaga cruenta	97,3	1	0	0	0	0	0	0	0	0	0	1	
AM406395		Proteobacteria	100	AY043376	Moraxella osloensis	99,5	1	0	0	0	0	0	0	0	0	0	1	
AM406405		Proteobacteria	98	AF498721	Bacterium Ellin339	99,3	1	0	0	0	0	0	0	0	0	0	1	
AM406415		Proteobacteria	100	AY463198	Comamonas sp. B0156	100	1	0	0	0	0	0	0	0	0	0	1	
AM406430		Proteobacteria	100	AF041446	Bradyrhizobium sp. 'SH 283012'	99,5	1	0	0	0	0	0	0	0	0	0	1	
AM406442		Proteobacteria	30	DQ057476	Bacterium ic1379	85,6	1	0	0	0	0	0	0	0	0	0	1	
AM406454		Proteobacteria	64	CP000251	Anaeromyxobacter dehalogenans 2CP-C	80,7	1	0	0	0	0	0	0	0	0	0	1	
AM406483		Proteobacteria	100	AY612302	Beta proteobacterium ASRB1	99,2	1	0	0	0	0	0	0	0	0	0	1	
AM406477		TM7	94	CR954246	Pseudoalteromonas haloplanktis str. TAC125	79,1	1	0	0	0	0	0	0	0	0	0	1	
AM275676		Verrucomicrobia	100	AY271254	Akkermansia muciniphila strain Muc	98,4	10	1	0	0	1	6	0	1	0	0	1	
AM405172		Verrucomicrobia	100	AY271254	Akkermansia muciniphila strain Muc	96,5	2	0	0	0	0	1	1	0	0	0	0	
