# Supplementary figures and images for: Sequence analysis of percent G+C fraction libraries of human faecal bacterial DNA reveals a high number of Actinobacteria
Source: BMC Microbiol. 2009 Apr 8;9:68. doi: 10.1186/1471-2180-9-68 (PMC2679024; doi:10.1186/1471-2180-9-68)

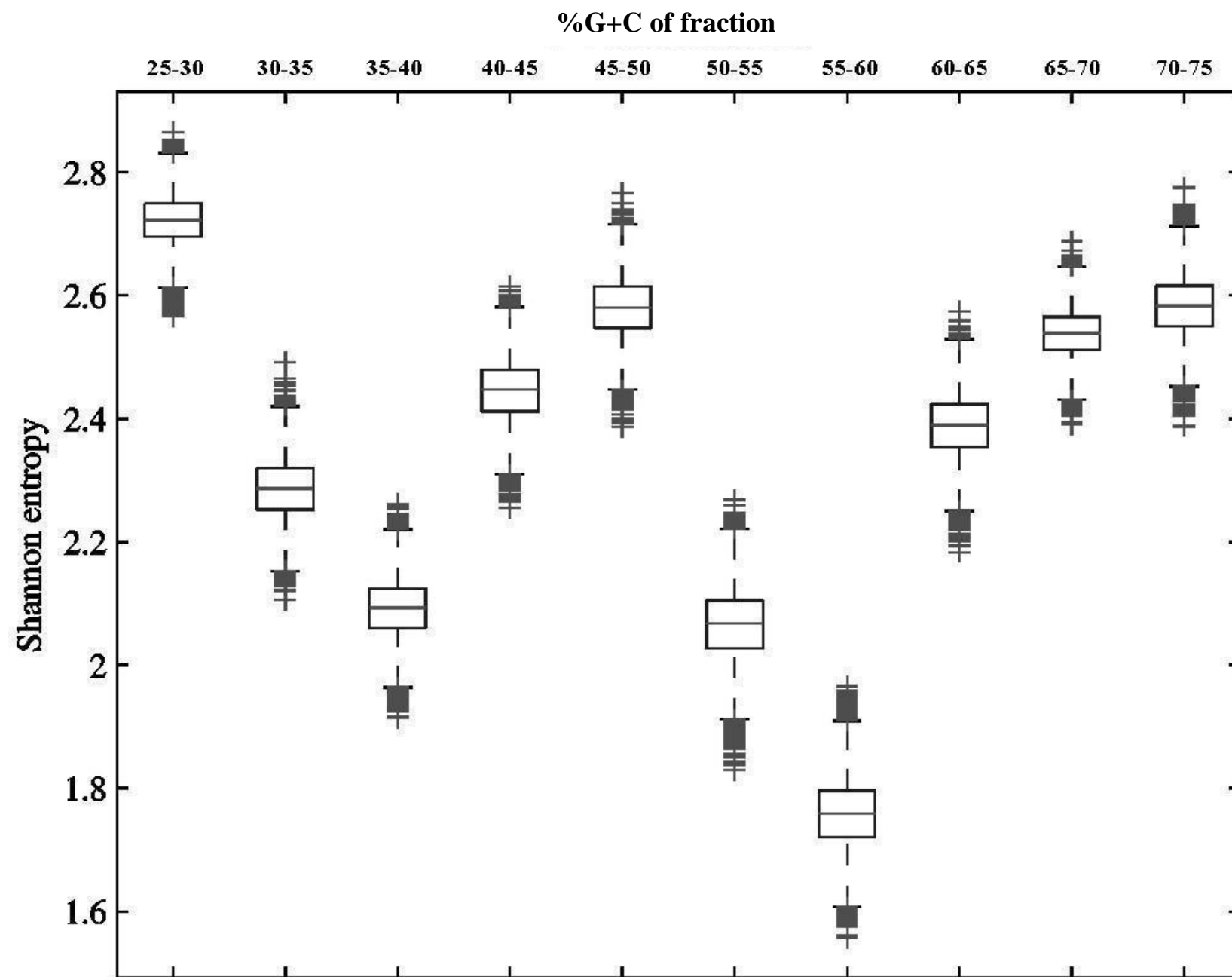

**Additional file 2** - Comparison of the %G+C clone library diversities using Shannon entropy.

Supplement: Additional File 2 — Comparison of the %G+C clone library diversities using Shannon entropy. The Shannon entropy values correlate with the amount and evenness of clusters or phylotypes in a community sample, but disregard the disparity between them. [file 1471-2180-9-68-S2.pdf]
