# Supplementary material for: Sequence analysis of percent G+C fraction libraries of human faecal bacterial DNA reveals a high number of Actinobacteria
Source: BMC Microbiol. 2009 Apr 8;9:68. doi: 10.1186/1471-2180-9-68 (PMC2679024; doi:10.1186/1471-2180-9-68)
Supplement: Additional File 3 — Clostridium cluster reference sequences. Unaligned Clostridium cluster reference sequences used in the phylogenetic analysis of sequence data. [file 1471-2180-9-68-S3.pdf]

Additional file 3 - *Clostridium* cluster reference sequences.

Accession no.	Species of origin	*Clostridium* cluster
X68182	*Clostridium acetobutylicum*	I
Y18176	*Clostridium disporicum*	I
X75272	*Clostridium grantii*	I
AF156796	*Clostridium peptidovorans*	I
AJ579907	*Clostridium sporogenes*	I
DQ978212	*Clostridium tetani*	I
M59113	*Clostridium tyrobutyricum*	I
M59094	*Clostridium histolyticum*	II
M59096	*Clostridium limosum*	II
X73448	*Clostridium proteolyticum*	II
X71854	*Clostridium termitidis*	III
X72870	*Clostridium stercorarium* subsp. *thermolacticum*	III
AJ305238	*Clostridium leptum*	IV
Y18187	*Clostridium orbiscindens*	IV
X81125	*Clostridium viride*	IV
Y18181	*Clostridium methylpentosum*	IV
L34618	*Eubacterium desmolans*	IV
AY169430	*Faecalibacterium prausnitzii*	IV
AB040495	*Oscillospira guilliermondii*	IV
DQ882649	*Ruminococcus bromii*	IV
L09165	*Thermoanaerobacter brockii*	V
AF028350	*Clostridium cocleatum*	VIII
X76743	*Clostridium thermoamylolyticum*	VIII
DQ449033	*Syntrophomonas wolfei* subsp. *methybutyica*	VIII
AY038995	*Megasphaera elsdenii*	IX
L09183	*Thermoanaerobacter cellulolyticus*	X
X73450	*Clostridium difficile*	XI
X77845	*Clostridium litorale*	XI
M59107	*Clostridium lituseburense*	XI
X80841	*Tissierella praeacuta*	XII
AY323523	*Peptostreptococcus micros*	XIII
AF125217	*Butyrivibrio fibrisolvens*	XIVa
DQ278862	*Clostridium aminophilum*	XIVa
M59090	*Clostridium coccoides*	XIVa
X73443	*Clostridium nexile*	XIVa
X71855	*Clostridium xylanolyticum*	XIVa
AY804151	*Eubacterium rectale*	XIVa
AY169414	*Lachnospira pectinoschiza*	XIVa
L14676	*Roseburia cecicola*	XIVa
AY169411	*Ruminococcus obeum*	XIVa
X76748	*Clostridium colinum*	XIVb
X77841	*Clostridium propionicum*	XIVb
M59120	*Eubacterium limosum*	XV
DQ440561	*Clostridium innocuum*	XVI
M59230	*Eubacterium biforme*	XVI
M23727	*Lactobacillus vitulinus*	XVII
X73440	*Clostridium ramosum*	XVIII
X73441	*Clostridium spiroforme*	XVIII
X77850	*Clostridium rectum*	XIX
AF543300	*Fusobacterium nucleatum*	XIX
