# Supplementary material for: Sequence analysis of percent G+C fraction libraries of human faecal bacterial DNA reveals a high number of Actinobacteria
Source: BMC Microbiol. 2009 Apr 8;9:68. doi: 10.1186/1471-2180-9-68 (PMC2679024; doi:10.1186/1471-2180-9-68)
Supplement: Additional File 4 — Reference sequences from the European ribosomal RNA database [56]. Reference sequences aligned according to their secondary structure and used in the phylogenetic analysis of sequence data. [file 1471-2180-9-68-S4.pdf]

Additional file 4 - Reference sequences from the European ribosomal RNA database (Wuyts *et al.*, 2004).

Phylum	Accession no.	Species of origin
Acidobacteria	D26171	*Acidobacterium capsulatum*
Actinobacteria	X79224	*Actinomyces birnadii*
Actinobacteria	S44206	*Atopobium parvulum*
Actinobacteria	M58729	*Bifidobacterium adolescentis*
Actinobacteria	D86183	*Bifidobacterium dentium*
Actinobacteria	M58739	*Bifidobacterium longum*
Actinobacteria	D86187	*Bifidobacterium pseudocatenulatum*
Actinobacteria	AB011816	*Collinsella aerofaciens*
Actinobacteria	X79048	*Coriobacterium glomerans*
Actinobacteria	AF079507	*Denitrobacterium detoxificans*
Actinobacteria	AJ009989	*Propionibacterium freudenreichii*
Actinobacteria	AF181690	*Rhodococcus* sp.
Actinobacteria	AF101240	*Slackia exigua*
Actinobacteria	AF101241	*Slackia heliotrinreducens*
Bacteroidetes	X83946	*Bacteroides fragilis*
Bacteroidetes	L16489	*Bacteroides thetaiotaomicron*
Bacteroidetes	AB003403	*Prevotella ruminicola*
Firmicutes	AF155952	*Bacillus cereus*
Firmicutes	M59090	*Clostridium coccoides*
Firmicutes	AF072474	*Clostridium difficile*
Firmicutes	Y18184	*Clostridium indolis*
Firmicutes	AF262239	*Clostridium leptum*
Firmicutes	AF067965	*Clostridium methoxybenzovorans*
Firmicutes	M59103	*Clostridium perfringens*
Firmicutes	X75908	*Clostridium spiroforme*
Firmicutes	X81125	*Clostridium viride*
Firmicutes	D14148	*Coprococcus eutactus*
Firmicutes	L34619	*Dorea formicigenerans*
Firmicutes	AB012212	*Enterococcus faecalis*
Firmicutes	M59230	*Eubacterium biforme*
Firmicutes	L34627	*Eubacterium rectale*
Firmicutes	X85022	*Fusobacterium prausnitzii*
Firmicutes	M58802	*Lactobacillus acidophilus*
Firmicutes	U95028	*Megasphaera elsdenii*
Firmicutes	L14676	*Roseburia cecicola*
Firmicutes	AF104839	*Ruminococcus albus*
Firmicutes	X85099	*Ruminococcus bromii*
Firmicutes	L76601	*Ruminococcus obeum*
Firmicutes	X85101	*Ruminococcus obeum*
Firmicutes	D14137	*Ruminococcus torques*
Firmicutes	AF104673	*Streptococcus intermedius*
Firmicutes	X84005	*Veillonella parvula*
Proteobacteria	AL139075	*Campylobacter jejuni*
Proteobacteria	Z83204	*Escherichia* coli
Proteobacteria	M59155	*Hafnia alvei*
Proteobacteria	X67024	*Pseudoalteromonas haloplanktis*
Verrucomicrobia	AF027005	unidentified *Verrucomicrobium*

Reference

Wuyts J, Perriere G, Van De Peer Y: **The European ribosomal RNA database.** Nucleic Acids Res 2004, **32(Database issue):**D101-3.
